# Strong Substrate–Adsorbate Interactions Direct the Impact of Fluorinated N-Heterocyclic Carbene Monolayers on Au Surface Properties

**DOI:** 10.1021/acsami.4c12514

**Published:** 2024-11-18

**Authors:** Iris Berg, Rajarshi Mondal, Joshua M. Sims, Tzipora Ben-Tzvi, Linoy Lahav, Barak Friedman, Carine Michel, Zackaria Nairoukh, Elad Gross

**Affiliations:** † Institute of Chemistry, The Hebrew University, Jerusalem 91904, Israel; ‡ The Center for Nanoscience and Nanotechnology, The Hebrew University, Jerusalem 91904, Israel; § ENSL, CNRS, Laboratoire de Chimie UMR 5182, 46 allée d’Italie, F69364 Lyon, France

**Keywords:** NHCs, SAMs, monolayers, coatings, fluorinated coatings

## Abstract

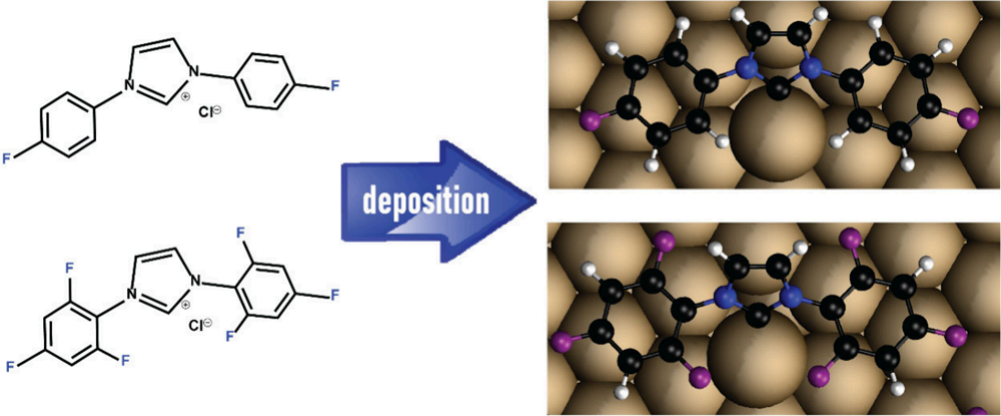

Fluorinated self-assembled monolayers (SAMs) have been utilized in a variety of applications such as transistors and optoelectronic devices. However, in most SAMs the fluorinated groups could not be positioned in high proximity to the surface due to steric effects. This limitation hinders the direct analysis of the impact of the fluorination level on surface properties. Herein, fluorinated aromatic N-heterocyclic carbenes (NHCs), with 1–5 fluorine atoms, were self-assembled on a gold substrate. These NHCs enabled the positioning of fluorinated groups in high proximity to the metal surface to identify the influence of the fluorination level on surface properties. Experimental measurements and theoretical calculations identified that all fluorinated NHCs formed SAMs and adopted a flat-lying adsorption configuration while anchored to the metal surface via Au adatom. A higher fluorination level induced a stronger interaction of the fluorinated side groups with the Au surface. The stronger interaction and surface proximity of the fluorinated side groups deteriorated the overall binding energy of the NHC due to the less-optimized adsorption geometry of the carbene carbon. Ultraviolet photoelectron spectroscopy measurements revealed that fluorinated NHC monolayers lowered the surface work function by up to 1 eV and induced an increase of 15–20° in the water contact angle. The impact on surface properties did not vary according to the fluorination level of NHCs, and similar values were measured for NHC with 1–5 fluorine atoms. It is therefore identified that dominant adsorbate–substrate interactions between the fluorinated side groups and the Au surface quenched the distinct impact of the fluorination level on surface functionality.

## Introduction

Self-assembled monolayers (SAMs) on surfaces are key elements for selectively modifying surface properties and therefore have been utilized for building devices for various applications,^1,2^ including organic electronics, photovoltaics, antifouling, and sensors.^3−5^ The broad range of applications was enabled due to the choice of both anchoring and side groups, which have a direct impact on the binding properties and the induced surface properties of the underlying substrate.

Specifically, SAMs containing fluorine groups have drawn significant attention due to the high electronegativity and low polarizability of the fluorine atoms. It was demonstrated that fluorinated monolayers can tune the physical properties of surfaces, including friction, wettability of various solvents, capacitance, and work function.^6,7^ However, the widely explored linear polyfluorinated thiols with more than six CF_2_ groups were found to be toxic and recently banned in several industrial countries,^8^ giving rise to the need for sustainable alternatives, such as shorter thiolate chains.^9^ Other alternatives include CF_3_ terminated aliphatic chains,^10^ branched fluorinated amphiphiles^11^ as well as polyfluorinated aromatic rings.^12^ Among these groups, polyfluorinated aromatic rings are of particular interest due to their wide use in organic field effect transistors (OFETs), optoelectronic devices, and molecular diodes.^13,14^ In these devices, the organic molecules were mostly used to control the work function and charge carrier dynamics of the substrate.^15,16^

The performance of the above-mentioned devices was shown to be influenced bythe binding mode and structure of the monolayers.^17,18^ Therefore, the incorporation of metal surfaces coated with fluorinated SAMs in industrial and medical applications requires systematic in-depth characterization of the properties of the monolayer structure and its effect on the metal surface. While such characterization was conducted for various fluorinated SAMs, it was mostly limited to thiol-based platforms.^19,20^

This platform, although simple to use, has a few weak points. Due to the lability of the Au–S bond, thiol-based monolayers suffer from limited stability even under ambient conditions.^21^ In addition, the fluorinated molecules in the thiol-based monolayer cannot be positioned in high proximity to the surface, and thus, the direct analysis of fluorination impact on surface properties cannot be determined.

N-Heterocyclic carbenes (NHCs) are known to form thermally stable SAMs on coinage metals, and particularly on gold substrates.^22,23^ Their high affinity toward coinage metals, as well as their high thermal and chemical stability, mark them as a plausible alternative to thiol-based SAMs. In contrast to the Au–S bond, which is cleaved upon exposure to 100 °C under ambient conditions,^24^ NHC-based monolayers on gold have demonstrated improved thermal stability.^22^

In addition, the NHC molecules provide a versatile platform, and a variety of side groups can be installed as nitrogen substituents in relatively simple synthetic steps.^25−27^ Their unique structure leads to an exceptional advantage of NHC-based functional SAMs: The proximity of the functional side groups to the surface maximizes the interaction between the groups and the surface, enabling the investigation of their mutual influence on each other.^28,29^ For example, it was recently demonstrated that aromatic fluorinated NHC monolayers can be incorporated as an organic field-effect transistor and the modified transistor showed superior performance compared to devices without the modification, attributed to improved carrier mobility.^30^ In addition, fluorinated groups were also used as a spectroscopic handle to study the self-assembly of NHCs on metal oxides^31^ and mesoionic carbenes on gold.^32^ The above-mentioned studies demonstrated the feasibility and impact of NHC fluorination and call for a methodical analysis of the influence of NHC fluorination degree on surface properties.

**Scheme 1:**
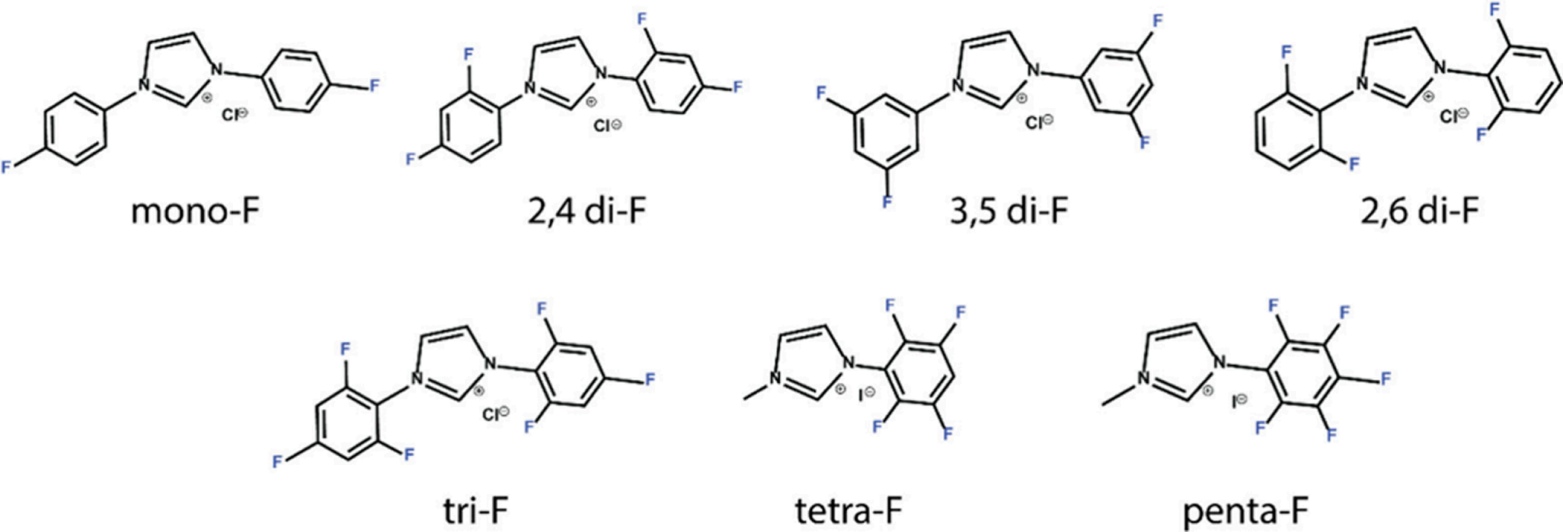
Molecular Structures of Fluorinated Imidazolium Salts, Which Were Utilized as Precursors for Fluorinated-NHC SAMs

Herein, we systematically analyzed the impact of the fluorination level on monolayer properties and substrate functionality. For this purpose, we have synthesized seven NHC-precursors bearing various numbers of fluorine substitutions, which range from one to five, as illustrated in Scheme 1, and deposited them on Au(111) single crystal. The variations in the fluorination degree enabled us to identify the effect of fluorine substituents on the adsorption behavior and its influence on surface properties.

It was determined that all fluorinated NHCs formed a monolayer on the Au surface. Interestingly, the strong interactions between the Au surface and the fluorinated phenyl rings quenched the impact of the fluorination level on surface properties, and therefore, a comparable impact was measured for all fluorinated SAMs. While the NHC backbone was thermally stable up to 200 °C for most molecules, partial decomposition of the fluorinated substituents was identified due to strong substrate–adsorbate interactions, demonstrating the crucial role of surface–adsorbate properties in the self-assembly pattern.

## Results and Discussion

The fluorinated imidazolium salts were synthesized according to published procedures. Mono-F-Cl^–^, 2,4 di-F-Cl^–^, 3,5 di-F-Cl^–^, 2,6 di-F-Cl^–^, and tri-F-Cl^–^ were synthesized^33,34^ in a one-pot process and isolated as chloride salts. Tetra-F-I^–^ and penta-F-I^–^ were isolated as iodide salts by a two-step synthetic technique.^35^ Due to synthetic challenges, the fluorination in tetra-F-I^–^ and penta-F-I^–^ salts was performed on one side of the molecule, thus yielding asymmetric structures. The isolated (3,5 di-F-Cl^–^, 2,6 di-F-Cl^–^, tetra-F-I^–^, and penta-F-I^–^) imidazolium salts were fully characterized by multinuclear NMR spectroscopy. NMR of mono-F-Cl^–^, 2,4 di-F-Cl^–^, and tri-F-Cl^–^ matched with previously reported data.^33^ NMR data of 3,5 di-F-Cl^–^, 2,6 di-F-Cl^–^, tetra-F-I^–^, and penta-F-I^–^ were measured in DMSO-d^6^ (Figures S1–S12).

**Figure 1:**
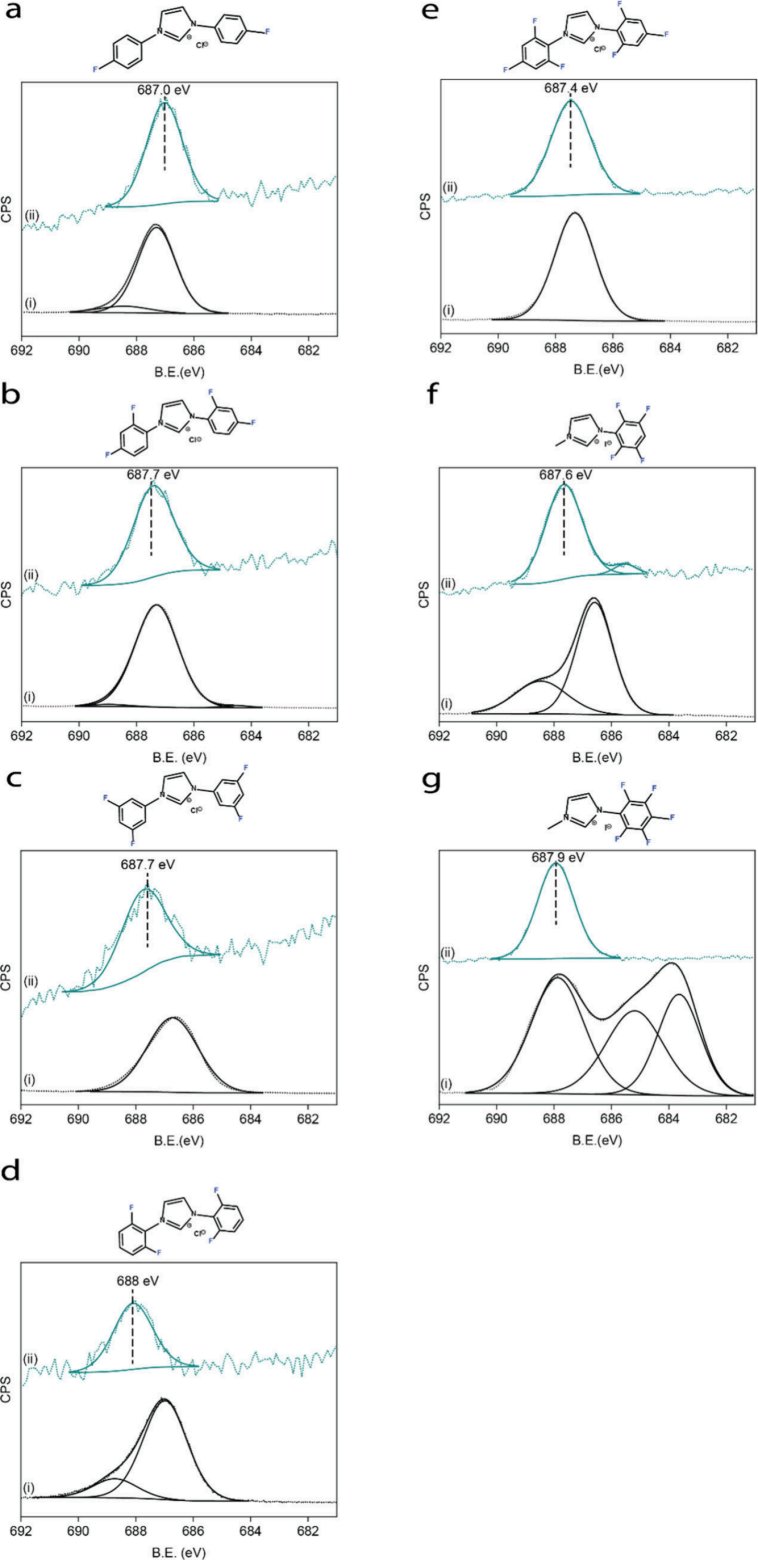
F 1s XPS signals of precursor salt (i) and F-NHCs monolayers (ii) on Au(111): (a) monofluorinated, (b) 2,4 difluorinated, (c) 3,5 difluorinated, (d) 2,6 difluorinated, (e) trifluorinated, (f) asymmetric tetrafluorinated, and (g) asymmetric pentafluorinated NHCs.

F 1s X-ray photoelectron spectroscopy (XPS) dataof the imidazolium salt precursors are shown in Figure 1a–g, spectra i. A dominant peak was detected at 686.5–687.5 eV for imidazolium salts with one, two, and three fluorine atoms (Figure 1a–e, spectra i). The asymmetric precursor molecules exhibited a more complex spectral pattern, with two peaks for the tetra-F precursor and three peaks in the spectrum of penta-F (spectra i in Figure 1f, g, respectively), as expected based on their asymmetric structure.

The imidazolium salts were deprotonated by their exposure to an inorganic base (potassium tert-butoxide), and F-NHCs were self-assembled on Au(111) single crystal to form F-NHC monolayers (see Experimental Section for details). F 1s XP-spectra of the F-NHCs monolayers (Figure 1a–g, spectra ii) reveal a common single feature for all molecules, positioned between 687.0 and 688.0 eV. The peak was correlated to the presence of the C–F bond in high proximity to the Au surface^36−38^ and is in agreement with the binding energy value measured for trifluorinated NHC on Au.^30^ Some of the molecules showed an additional, minor peak that was centered at 689 eV, which could be attributed to a minor contaminant with high fluorine density.^39^

Generally, higher binding energy values were measured with an increasing number of fluorine atoms on the aromatic ring. The binding energy was 687.0 eV for the mono-F NHC monolayer and increased to 687.9 eV for the penta-F NHC monolayer. This shift can be attributed to the increasing number of electron-withdrawing fluorine groups. It was previously identified that F 1s XP-spectra of monolayers with high density of fluorine atoms (e.g., CF_3_ groups) are characterized with higher binding energy, of up to 688 eV.^40^

Notably, the F 1s XP-spectra of all the surface anchored NHC monolayers wereconstructed of one main peak, although the F 1s XP-spectra of tetra-F and penta-F precursor molecules were comprised of more than one Gaussian. The differences in the peak pattern between the precursor and the anchored molecules, which was obtained for tetra-F and penta-F NHC monolayers, may imply an electronic change induced by interaction of the fluorinated-arynes and the Au surface. Such an interaction can lead to degeneration of the electronic properties of the fluorine atoms in the molecule. The appearance of a single peak for surface-anchored asymmetric structures is consistent with previous observations of a single F 1s peak for asymmetric aromatic polyfluorinated monolayers.^41^ The F 1s XPS spectrum of tetra-F NHC monolayers exhibited an additional small peak, positioned at 685 eV (Figure 1f) and correlated to a semi-ionic fluorine species,^38^ possibly due to strong interaction with the gold substrate.

N 1s XP-spectra (Figure S13) of the surface-anchored molecules further confirmed the successful deposition of fluorinated NHCs on the surface. In the XPS spectra of the precursor molecules (Figure S13, spectra i), the main peak was centered around 401–403 eV, and an additional peak was observed at 399–400 eV. The additional peak may be attributed to the presence of synthesis impurities.^30^ Upon surface deposition, the main peak was positioned at 401 eV, and an additional side peak was probed at a lower binding energy of 398–399 eV, as observed in previous work for fluorinated NHCs.^30^ In the case of asymmetric molecules, the additional peak may be explained by the two distinct nitrogen atoms of the molecules. In the symmetric molecules, however, the additional peak may reveal the presence of physisorbed impurities.^30^

C 1s XPS spectra were measured but did not provide additional information, as the signal originating from adventitious carbon was in the same order of magnitude as the carbon signal of the monolayer (Figure S14). The Au 4f XPS signal was measured as well, and the signal pattern did not change upon deposition of F-NHCs (Figure S15).

As the monolayers were prepared from precursors containing halide counterions, XPS spectra of the respective halides were measured to assess the concentration of counterions following F-NHC deposition and their potential impact on monolayer formation. For the monolayers prepared from precursors with chlorine as counterion, no traces of chlorine were detected on the surface following the cleaning procedure. In the case of monolayers prepared from iodine-containing precursors, however, traces of iodine were detected by XPS (Figure S16), although in trace amounts of 0.36% for tetra-F NHCs and 0.37% for penta-F NHCs, which is an order of magnitude lower than the measured concentration of fluorine and nitrogen.

Quantitative analysis of the XPS peaks was done based on F-NHC deposition on Au(111) single crystal (Table S1). The N 1s/Au 4f values that were measured for the F-NHCs were used for the calculation of F-NHC surface density. In addition, the N 1s/Au 4f values were compared to the surface density of nitro-functionalized NHCs, which were quantified by XPS and NO_2_ electroreduction (see Table S1 for calculation details).^42^ The calculation revealed that the surface density of the symmetric fluorinated NHCs was 5–7 × 10^–12^ mol·cm^–2^ and 2–6 × 10^–11^ mol·cm^–2^ for the two different calculation routes, which is comparable to that of nitro-functionalized NHCs. The surface density of the asymmetric penta- and tetra-F NHC monolayers was 2-fold higher than the density of the symmetric molecules, correlated to their smaller steric footprint. Control experiments of physisorbed precursors did not show any F 1s XPS signal, demonstrating the crucial impact of the carbene group in anchoring the F-NHC molecule on the surface (Figure S17).

Cyclic voltammetry (CV) measurements in the presence of [Fe(CN)_6_]^−3^/[Fe(CN)_6_]^−4^ redox couple were conducted following deposition of mono-, tri-, and penta-F NHCs to probe the presence of the monolayers on the surface and assess the surface coverage (Figure S18). The CV validated the relatively low surface density of the mono-F and tri-F NHCs, as little to no surface passivation was observed. The penta-F NHC monolayer, however, was denser and partially passivated the surface, in agreement with its lower steric footprint and higher surface density, as calculated above.

Polarization modulation-infrared reflection absorption spectroscopy (PM-IRRAS) measurementsof F-NHC monolayers on Au films (Figure S19) identified red shifts in the C–F region (1100–1500 cm^–1^) following surface-anchoring,^43,44^ attributed to strong interactions between the fluorinated aromatic rings and the Au surface that led to weaker C–F bonds.^38^

**Figure 2:**
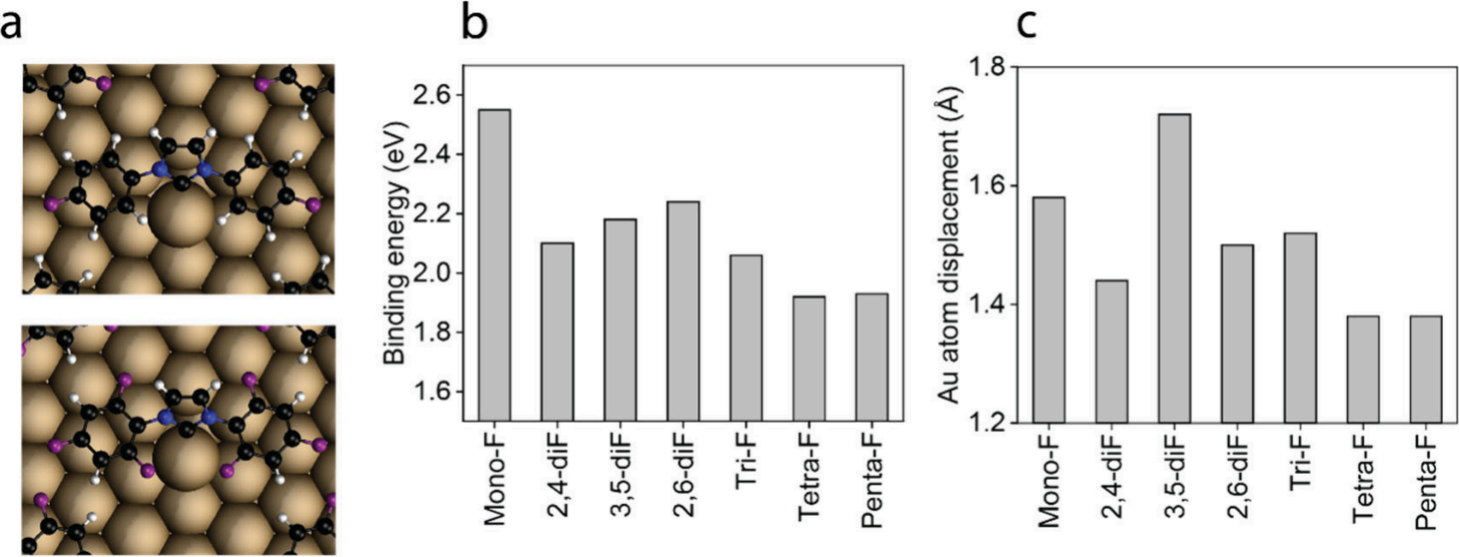
(a). Optimized structures of mono-F and tri-F NHCs on Au(111). Strong C–Au interaction induced vertical displacement of a Au atom, while the phenyl rings were positioned in parallel to the Au surface. (b) Adsorption energy (numbers are gives in absolute value) and (c) Au adatom displacement values for the different F-NHCs.

DFT calculations were performed to identify the adsorption energies and adsorption geometries of fluorinated NHCs. The binding energy and the optimized adsorption geometry of F-NHCs on the Au(111) surface were evaluated using a coverage close to the experimental one (Figure 2 and Table S2). All F-NHCs adopted a similar adsorption geometry with respect to the gold substrate, with the carbene ring tilted with respect to the surface normal and the fluorinated phenyl rings flat-lying on the surface (Figure 2a). A similar adsorption geometry was obtained for nitro functionalized NHCs^45^ and for NHCs with bulky side groups.^46^ The DFT calculations therefore rationalize the high similarity in the F 1s XPS signal of the various fluorinated NHCs (Figure 1), which is correlated to strong substrate–adsorbate interactions that were induced by the high surface proximity of fluorinated aromatic rings.

The adsorption energy values decreased with an increase in the number of fluorine groups (Figure 2b). The values varied from −2.55 eV for the monofluoro carbene to −1.92 eV for the tetrafluoro carbene. These values are similar to the values that were calculated for NHCs with isopropyl groups (−2.85 eV).^46^ In addition, the DFT calculations identified a displacement of the Au surface atom on which the NHC was anchored. The displacement of the Au adatom was in line with the calculated adsorption energy of the molecules (Figure 2c). The strong surface binding of fluorinated NHCs may generate an adatom, as identified for other NHC-based monolayers on Au.^45,47^ The impact of the fluorination degree on binding energy and Au atom displacement is correlated to the interaction of the side group with the Au surface. Strong interaction of fluorinated side groups with the surface induced a higher surface proximity that quenched the Au adatom displacement and thus lowered the overall adsorption energy, which is mostly correlated to the interaction of the carbene carbon with the metal surface and is maximized on an adatom. Thus, a higher fluorination degree induced stronger surface interaction of the fluorinated wingtip groups, which led to higher surface proximity, lowered the Au adatom displacement, and thus lowered the carbene binding energy. It is hypothesized, based on recent works,^48,49^ that the variation in the Au atom displacement value for 3,5-diF was induced due to fluorination at the meta position that can cause a decrease in the sigma donation in carbene carbon and an increase in pi electron acceptance from the metal via back-donation. The monolayer thickness was determined by using ellipsometry. The measured thickness was 0.4, 0.5, and 0.4 ± 0.2 nm for mono-, tri-, and penta-F NHCs monolayers, respectively, in line with a calculated flat-lying adsorption geometry.^50^

**Figure 3:**
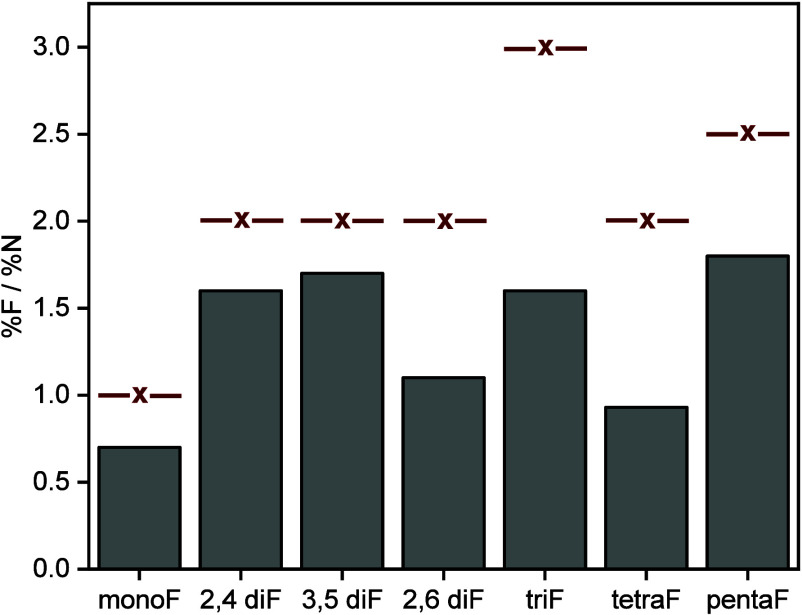
Atomic percentage of fluorine-to-nitrogen ratios for F-NHC monolayers (gray-colored bars), as determined by XPS measurements, relative to the expected ratio (red-colored lines).

The atomic fluorine-to-nitrogen ratio was calculated by analysis of XPS signals (Figure 3). For most of the fluorinated molecules, the measured ratio (gray colored columns in Figure 3) was lower than the expected ratio by 20–30% based on the molecular structure (marked by red-colored marker in Figure 3). The measured ratio in most of the salt precursors was closer to the expected value (Figure S20). These variations can indicate dissimilarity in the cross section of the two elements due to strong surface interactions and higher proximity of the fluorinated groups, in comparison to nitrogen.^51^ Notably, the measured fluorine-to-nitrogen atomic ratio for 2,6 di-F NHCs, tri-F NHCs, and tetra-F NHCs was lower by 50% than the expected ratio, which can indicate that fragmentation was facilitated on the Au surface. As detailed below, the nature of the fragmentation was identified via DFT calculations as bond dissociation between the imidazole ring and the fluorinated side ring.

**Figure 4:**
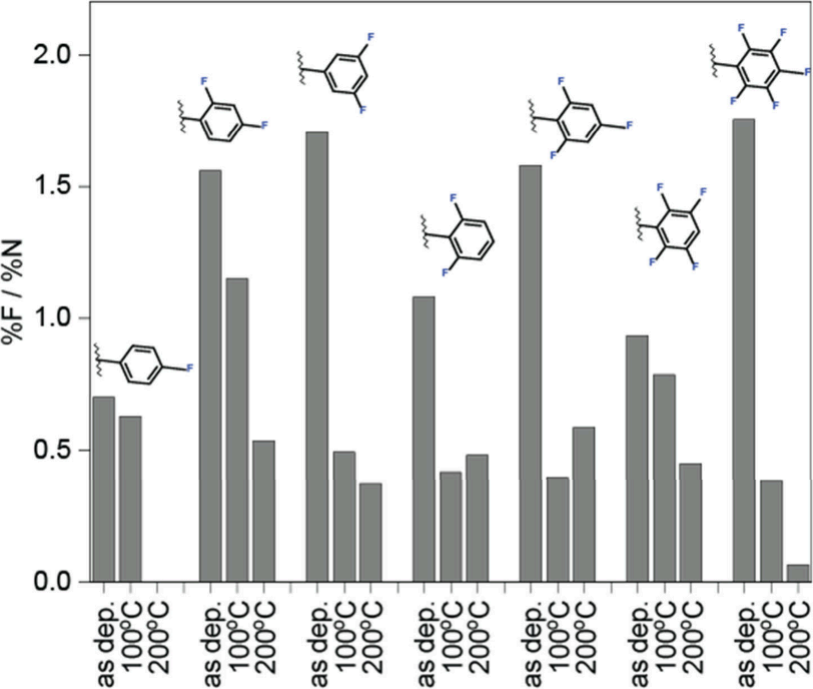
Atomic percentage of fluorine-to-nitrogen ratios for F-NHC monolayers at room temperature (rt) and after annealing to 100 and 200 °C. The atomic ratio was calculated based on XPS measurements.

In order to evaluate the thermal stability of the various F-NHCs and their surface interactions, XPS measurements were conducted after exposure of the F-NHCs SAMs to elevated temperatures (Figures S21 and S22). Analysis of the fluorine-to-nitrogen ratio for the different F-NHCs following thermal treatments is shown in Figure 4, to identify surface dissociation and fragmentation routes. 2,6 di-F NHCs, tri-F NHCs, and tetra-F NHCs showed partial fragmentation already at room temperature. Other F-NHCs showed fragmentation only after annealing to 100 °C, while 3,5 difluorinated NHCs were stable at 100 °C and fragmented at 200 °C. The high thermal stability of 3,5 difluorinated NHCs may be attributed to stabilizing resonance effects.

Notably, the decrease in the fluorine atomic ratio was more significant compared to the decrease of the nitrogen signal at elevated temperatures (Figure 4). This observation may imply that fragmentation and partial decomposition occurred on the surface. Analysis of the decomposition process was achieved by a detailed DFT study of the dissociation routes for tetra-F NHC monolayer, which is prone to fragmentation according to the measured F/N ratio (Figure 4).

The possible fragments of the C–H, C–F, and C–N scissions were optimized on the Au surface, and then, the corresponding XPS spectra were simulated (full results are presented in Tables S3 and S4). Using this approach, it is possible to eliminate decomposition by C–H or C–F bond dissociation, as the predicted XPS spectra of the resulting fragments do not match the experimental XPS spectra. C–H bond dissociation will not affect core level bonding energies of the fluorine atoms in a way that can lead to multiple bands in F-XPS spectra, and C–F bond dissociation will lead to a shift of ∼5 eV in the bonding energy, which was not observed experimentally. The experimental F 1s XPS data are, however, compatible with the decomposition by dissociation of C–N bond. The F 1s XPS data at 200 °C indicate that additional fragmentation occurred at this stage, which can include the dissociation of additional C–N or C–H bonds.

**Figure 5:**
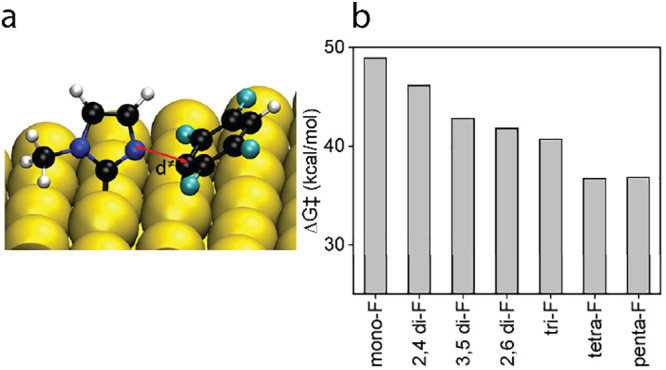
(a) Optimized transition state for C–N bond cleavage in tetra-F NHC (*d*^⧧^ = 2.03 Å); (b) transition state energies for the various F-NHCs. For more information, see Table S5.

The variation in the thermal stability of F-NHCs can be further explained by calculating the activation energy barrier for breaking the C–N bond, which is expected to be the first step in the fragmentation process, as identified based on F 1s XPS data and theoretical analysis. DFT simulations analyzed the C–N bond scission in fluorinated NHCs (Figure 5) with the fluorinated phenyl ring tilted to forma C–Au bond. The corresponding activation barriers (Table S5) generally correlate with the experimental observations. The NHCs that partially decomposed at room temperature have C–N bond breaking activation barriers lower than 42 kcal/mol, and the NHCs that decompose at 100 °C have C–N bond breaking activation barriers greater than 43 kcal/mol. The thermal dissociation pattern further demonstrates the strong surface anchoring of NHCs, which facilitates the dissociation of the C–N bond over ligand detachment from the Au surface.

While the absolute values of these activation barriers suggest that these reactions will not occur in the relevant temperature range, several factors could lead to lower activation energy barrier, in particular the possibility that the reaction might not occur on a pristine 111 surface but on steps on the surface, which can lead to higher reactivity^52^ and easier bond-breaking.^53,54^ While the absolute values of the activation barriers are undoubtedly lower on more active sites, the general trend seen in Figure 2 still holds true, giving further evidence that the NHC fragmentation mechanism on Au(111) is initiated by dissociation of the C–N bond and continues with desorption of the volatile species (possibly fluorinated phenyl rings), as also experimentally evidenced by a lower than expected concentration of fluorine on the surface (Figure 4).

AFM topography analysis of F-NHC SAMs on gold films before and after annealing were conducted to probe the influence of NHC deposition and annealing on the surface topography (Figure S23). The AFM scans revealed that surface topography and roughness were slightly varied following NHC deposition, mainly correlated to the presence of base or solvent residues on the surface, which were removed from the surface following annealing.

**Figure 6:**
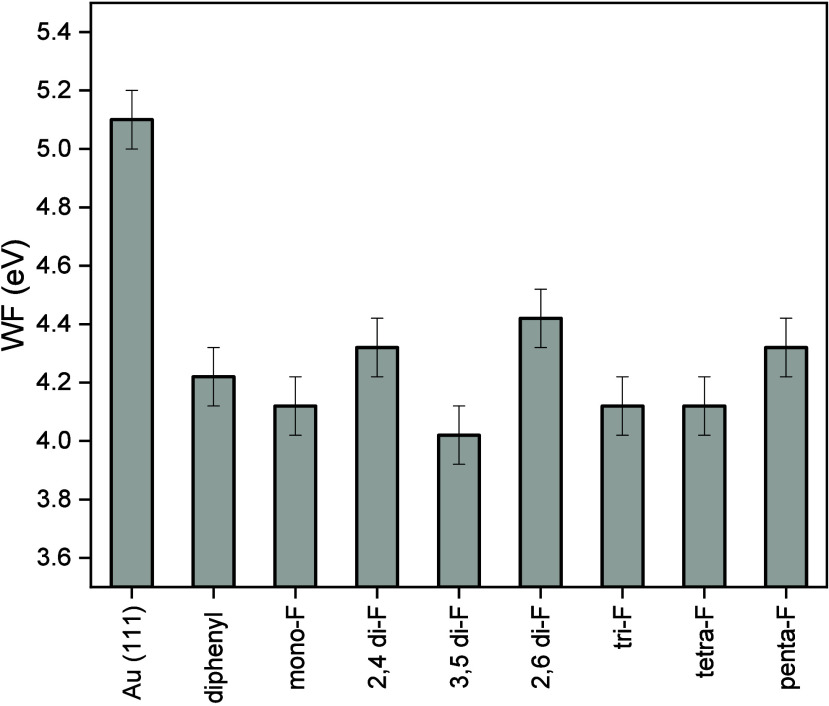
Work function values for F-NHC SAMs and diphenyl-NHC on Au(111).

The influence of the fluorination level in F-NHCs on the properties of the gold substrate was assessed by ultraviolet photoelectron spectroscopy (UPS) and water contact angle measurements. The measured work function values of F-NHCs SAMs on Au(111) are presented in Figure 6 (see Figure S24 for the UPS spectra). The values of the work function measured on the bare Au(111) and on a surface coated with a nonfluorinated NHC that is functionalized with nonfluorinated phenyl substituents (termed “diphenyl”, see the SI for synthetic details) are presented as reference points. SAMs of F-NHCs lowered the work function (WF) by up to 1.1 eV, while a decrease of 1 eV was measured for diphenyl-NHC, which is not functionalized with fluorinated substituents.

The effect of the fluorinated NHCs on work function values was similar to the nonfunctionalized NHC. In addition, the effect of the different molecules on the work function was rather similar and did not reveal a dominant impact of the fluorination degree or the fluorination pattern. This result nicely correlated with the F 1s XPS data, which showed similar binding energy for all F-NHC, indicative ofstrong substrate–adsorbate interactions that quenched the impact of the fluorination level.

Fluorinated aromatic thiols modified the electronic properties of the underlying substrate.^55,56^ Mono- and penta-fluorinated benzenethiols were shown to increase the WF of gold by 0.4–0.7 eV,^56^ in accordance with the direction of the dipole moment of the molecules, assuming an upright adsorption geometry. However, in the case of tetra- and penta-fluorinated aromatic thiols, the effect was shown to be mainly dependent on the molecular arrangement, rather than the number of fluorine atoms on the aromatic ring.^55^ When examining other electronic properties, such as the electron transport properties, it was observed that alternating the positions of the fluorine groups in difluorinated aromatic thiols did not lead to a significant change in the measured properties.^41^ These observations support the general trend observed in this work, with relatively minor variations in the measured work function values upon changing the fluorination pattern and number of fluorine atoms . Nonfluorinated carbene-based monolayers lowered the work function of gold surfaces^45,57−59^ by 1.1–2.0 eV, a larger effect compared to the values measured in this work and correlated to the higher surface density of these NHCs, which are characterized with smaller steric footprint.^57^

Water contact angle (CA) values of F-NHC SAMs on Au surfaces were measured (Figure S25). F-NHC monolayers changed the water contact angle by 15–20° relative to that of the bare Au. Higher fluorination degree did not lead to major changes in the contact angle. Moreover, the F-NHC-based monolayers did not increase the contact angle more than the reference NHC with phenyl substituents. The contact angle is affected by the molecular ordering of a monolayer, as well as by the surface dipole moment.^60,61^ In previous works, it was observed that in the case of aromatic thiols, substitution of one hydrogen atom for a fluorine or CF_3_ group had a minor effect of 1°–12° change in the static or advancing water contact angle relative to the nonsubstituted molecules.^61,62^ Substitution of five of the hydrogen atoms had a more significant effect of increasing the angle by ca. 30°.^61^ In this context, it is important to note that the described monolayers were found to be densely packed and adopt a vertical geometry, slightly tilted with respect to the surface normal, so that the fluorine atoms were located in an exterior position.

In the case of F-NHCs, the bulkier nature of the molecules can prevent the formation of densely packed monolayers. The relatively minor changes in the contact angle imply that the fluorine atoms do not point out of the surface or that their density is not sufficient for a significant influence. According to DFT calculations, the fluorinated phenyl rings adopted a flat-lying adsorption geometry. The common adsorption geometry, as well as the relatively low surface density of the fluorine atoms, may explain the similarity in contact angle values that were measured for the different monolayers.

Three of the molecules (2,6 di-F, tetra-F, and penta-F) lowered the contact angle by 10° compared to the bare gold. As discussed above, these three molecules showed partial decomposition during the deposition process (Figure 3), possibly due to high density of fluorine atoms and their repulsion from the surface. These structural features may lead to variations in their packing density in comparison to those of the other F-NHCs, which can explain the smaller influence on the contact angle. To conclude, the fluorinated molecules decreased the substrate work function and the water contact angle. There was, however, no dominant impact of the fluorination level on the magnitude of the effect on surface properties. The relatively low surface density of F-NHCs, along with the similarity in the absorption geometry and the resulting strong surface interactions, may explain the limited correlation between changes in surface properties and the degree of fluorination in F-NHC.

## Conclusions

The impact of fluorination level on surface properties was systematically studied by synthesizing seven fluorinated NHC molecules and analyzing their self-assembly pattern on Au(111) and their impact on surface properties. In all cases, the F-NHC monolayers adopted a flat-lying adsorption geometry and were attached to the surface via an adatom. Higher fluorination degree induced stronger interactions of the fluorinated side groups with the Au surface and lowered the adsorption energy of the carbene carbon with the Au surface. F-NHCs differed by their stability, whereas some of the molecules partially decomposed already upon deposition via C–N bond dissociation and loss of the fluorinated aromatic ring, while others exhibited higher thermal stability. F-NHC monolayers altered the Au work function by up to 1 eV and increased the water contact angle values of the Au substrate. However, these effects did not depend on the degree of fluorination, in accordance with strong surface interactions that were induced by the flat-lying adsorption geometry of the fluorinated side groups. This work therefore demonstrates the impact of F-NHC on surface properties but also shows the dominant role of adsorbate–substrate interactions, which quenched the effect of variations in the fluorination degree on surface properties.

## Experimental Section

### NHC Synthesis

#### Materials and Methods

All reagents, chemicals, and solvents were purchased from different chemical vendors at the highest grade of purity. The solvents were used as reagent grade without any further purification. Unless otherwise stated, reaction temperatures are reported here as the temperature of the bath surrounding the vessel. Column chromatography was performed with Silicycle ultrapure silica gel P60. ^1^H, ^13^C{1H} NMR spectra were recorded on Bruker 500 or 400 spectrometers at room temperature with chemical shifts reported in parts per million (ppm) relative to the residual deuterated solvent or the internal standard tetramethylsilane. The fluorinated and nonfluorinated imidazolium salts **(Mono-F-Cl**^**–**^**, 2,4di-F-Cl**^**–**^**, 3,5 di-F-Cl**^**–**^**, 2,6 di-F-Cl**^**–**^**, tri-F-Cl**^**–**^**, tetra-F-I**^**–**^**, penta-F-I**^**–**^**, diphenyl-Cl**^**–**^**)** have been synthesized following the same or modified published procedures.

##### Synthesis and Characterization Data for 3,5 di-F-Cl^–^, 2,6 di-F-Cl^–^, tetra-F-I^–^, penta-F-I^–^

Following a modified literature procedure,^34^ 3,5-difluoroaniline (0.258 g, 10 mmol) and paraformaldehyde (140 mg) was taken in a flask with toluene (5 mL). The mixture was refluxed until all paraformaldehyde was dissolved. The resulting solution was taken to 40 °C before glyoxal (40 wt % 0.58 mL) was added. Then the mixture was refluxed for 15 min before slow addition of chlorotrimethylsilane (2.5 mL, 20 mmol), and refluxing continued overnight. The resulting mixture was pumped and dried under vacuum to obtain a dark brown slurry. Silica gel column chromatography was performed using DCM:Methanol (9:1) as an eluent. White solid was obtained. Isolated yield: 0.624 g (38%).

^1^H NMR (500 MHz, DMSO-*d*^*6*^, 22 °C) δ 10.97 (br, 1H, C_imidazolium_–H), 8.74 (s, 2H, C_imidazolium_–H), 8.06 (br, 4H, C_Ar_–H), 7.62 ppm (m, 2H, C_Ar_–H); ^13^C NMR (126 MHz, DMSO-*d*^*6*^, 22 °C) δ 163.6 (d, *J* = 13 Hz), 161.7 (d, *J* = 13 Hz), 136.2 (t, *J* = 13 Hz), 136.1 (s), 121.6 (s), 106.2 (m), 105.6 ppm (t, *J* = 28 Hz); ^19^F NMR (471 MHz, DMSO-d^6^, 22 °C) δ −105.69 ppm (m).

##### 2,6 di-F-Cl^–^

Following a literature procedure **2,6 di-F-Cl**^**–**^ was synthesized.^34^ White solid. Isolated yield: 0.721 g (45%).

^1^H NMR (400 MHz, DMSO- *d*^*6*^, 22 °C): δ 10.37 (s, 1H, C_imidazolium_–H), 8.54 (s, 2H, C_imidazolium_–H), 7.83 (m, 2H, C_Ar_–H), 7.59 ppm (m, 4H, C_Ar_–H), ^**13**^C NMR (101 MHz, DMSO-*d*^*6*^, 22 °C): δ 157.2 (m), 154.7 (m), 141.2 (s), 133.5 (m *J* = 10 Hz), 125.0 (m), 113.3 (m), 113.1 (m), 112.2 ppm (t, *J* = 16 Hz). ^19^F NMR (376 MHz, DMSO-*d*^*6*^, 22 °C): δ −120.77 ppm (m).

##### Tetra-F-I^–^

Following a literature procedure^35^**tetra-F-I**^**–**^ was synthesized using 2,3,5,6-tetrafluoroaniline (0.825 g, 5 mmol), paraformaldehyde (140 mg), glyoxal (40% wt. 0.58 mL) and ammonium acetate (0.383 g, 5 mmol) in acetic acid (2 mL in total) and water (1 mL) at 70 °C for overnight stirring. Upon basification, a faint yellow oil was isolated, and it was used without further purification for the next step, where it was mixed with 2-iodomethane (10 mmol) in toluene (10 mL) and refluxed for 24 h. White solid. Isolated yield: 0.875 g (49%).

^1^H NMR (400 MHz, DMSO- *d*^*6*^ 22 °C): δ 9.69 (s, 1H, C_imidazolium_–H), 8.37 (s, 1H, C_Ar_–H), 8.18 (s, 1H, C_imidazolium_–H), 7.62 (s, 1H, C_imidazolium_–H), 4.04 ppm (s, 3H, -Me). ^13^C NMR (126 MHz, DMSO- *d*^*6*^, 22 °C): δ 146.4 (m), 144.5 (m), 142.5 (m), 140.6 (m), 138.9 (s), 124.8 (s), 123.9 (s), 114.9 (s), 109.1 (t, *J* = 24 Hz), 36.8 ppm (s). ^19^F NMR (376 MHz, DMSO- *d*^*6*^, 22 °C): δ −137.85 (m), −147.30 ppm (m).

##### Penta-F-I^–^

Following the synthesis of **tetra-F-I**^**–**^, **penta-F-I**^**–**^ was synthesized using 2,3,4,5,6-pentafluoroaniline (5 mmol). Yellow oil. Isolated yield: 0.793 g (42%).

^1^H NMR (400 MHz, DMSO- *d*^*6*^, 22 °C): δ 9.66 (s, 1H, C_imidazolium_–H), 8.14 (s, 1H, C_imidazolium_–H), 8.07 (s, 1H, C_imidazolium_–H), 4.02 ppm (s, 3H, -Me). ^13^C NMR (101 MHz, DMSO-*d*^*6*^, 22 °C): δ 206.5 (s), 143.5 (m), 141.5 (m), 139.1 (s), 138.3 (m), 136.4 (m), 124.9 (s), 123.9 (s), 110.8 (br m), 36.8 ppm (s). ^19^F NMR (376 MHz, DMSO-*d*^*6*^, 22 °C): δ −146.5 (m, *J* = 23), −150.37 (m, *J* = 23), −160.9 ppm (m, *J* = 23).

##### Diphenyl-NHC

A modified literature procedure^63^ was followed using 1 aniline (0.931 g, 10 mmol) and paraformaldehyde (150 mg, 5 mmol), glyoxal (0.58 mL, 40% wt., 5 mmol), HCl (1.7 mL, 3M) in toluene 10 mL. Silica gel column chromatography was performed using DCM:Methanol (9:1) as eluent. White solid was obtained. Isolated yield: 0.131 g (51%). NMR chemical shifts matched the reported values.^8^

### Deposition and Characterization

Gold films (100 nm thickness) were prepared by gold evaporation on a highly doped n-type Si wafer coated with a 10 nm thick Cr film. The samples were cleaned via a few sonication cycles in triply distilled water (TDW), acetone, and isopropanol.

Fluorine-functionalized imidazolium salt or dimethyl imidazolium salt was dissolved in THF (30 mM) in a glovebox and mixed with a THF solution of potassium tert-butoxide (60 mM) for 2 h for deprotonation and formation of the corresponding free carbenes. The freshly prepared solution with the fluorinated-NHCs was transferred through a syringe with a filter unit to a vial in which the Au-coated Si wafers or Au(111) single crystals were deposited. After 18 h, the surfaces were removed from the glovebox and rinsed three times with tetrahydrofuran, three times with distilled water, and two times with ethanol. The samples were then flushed with N_2_ for 5 min.

Tetrafluorobenzene and pentafluorobenzene control samples were prepared by diluting tetrafluorobenzene and pentafluorobenzene imidazolium salts in THF to a concentration of 100 mM, and drop casting the solution on clean Au surfaces. The excess solvent was dried in a hood at room temperature.

X-ray photoelectron spectroscopy (XPS) measurements were performed using a Kratos AXIS Supra spectrometer (Kratos Analytical) with an Al Kα monochromatic X-ray source (1486.6 eV). The XPS spectra were acquired with a takeoff angle of 90° (normal to analyzer), pass energy of 20 eV, and step size of 0.1 eV; the vacuum condition in the chamber was 2 × 10^–9^ Torr. The binding energies were calibrated according to the Au 4f XPS peak position (B.E. = 84.0 eV). Data were collected and analyzed using Casa XPS.

UPS measurements were performed using a Kratos AXIS Supra spectrometer (Kratos Analytical) with a He I α monochromatic UV source (21.22 eV). The UPS spectra were acquired under the same conditions as those of XPS, with a pass energy of 10 eV, step size of 0.025 eV, and an aperture of 110 μm.

Polarization-modulation infrared reflection absorption spectroscopy (PM-IRRAS) measurements were performed at room temperature under positive nitrogen pressure in a reflection–absorption cell (Harrick, Inc.) with a PM-FTIR spectrometer (PMA-50 coupled to Vertex 70. Bruker). 2048 scans were performed with a resolution of 4 cm^–1^ while using a mercury–cadmium–telluride (MCT) detector.

Contact angles were measured with a Ramé-Hart model 100 contact angle goniometer. The measurement was repeated three times for each sample, and the averaged values were reported.

The coated sample thickness was measured with an ellipsometer (FS-1 Multiwavelength Ellipsometer, Film Sense).

Tapping-mode AFM measurements were performed by using a nanoIR3 system (Bruker).

CV measurements were conducted with a potentiostat (Bio-Logic) using a three-electrode glass cell. Ag/AgCl (KCl 1M) was used as a reference electrode, and platinum wire was used as a counter electrode. The samples were immersed in 0.1 M KCl containing 10 mM [Fe(CN)_6_]^−3^ and 10 mM [Fe(CN)_6_]^−4^ (aqueous) during CV measurements.

### Computational Details

Periodic density functional theory as implemented in the Vienna Ab-initio Simulation Package (VASP)^64^ was used to approximate electronic energies at the GGA level using the Perdew–Burke–Ernzerhof (PBE)^65^ exchange–correlation functional with dDSc dispersion correction.^66^ Ion–electron interactions were treated using projector augmented wave (PAW)^67^ formalism with 645 eV cutoff for the augmentation charge, and plane-wave basis set was truncated at 400 eV. The p(4 × 4) Au(111) slabs were cleaved from the optimized bulk Au leading to a Au–Au distance of *a* = 2.93 Å, in good agreement with experimental values. All degrees of freedom were allowed to relax until all forces were less than 0.02 eV/AÅ except the 2 bottom layers of the Au slab. The cutoff for the electronic self-consistency cycle was set to 1 × 10^–6^ eV. All calculations for slab or slab–adsorbate systems used the Γ-point. A second-order Methfessel–Paxton smearing scheme was employed with a smearing width of 0.2 eV. Adsorption of NHCs led to strong displacement of Au.

Adsorption of an NHC on the Au surface leads to one of the Au atoms shifting upward above the surface. It would be possible to model this by adding a single Au atom on the surface in order to avoid the formation of a void due to this upward movement. However, in the study, it was preferable not to do this in order to avoid excessively complicating the reaction paths. Tests show that this has no impact on XPS calculations, and changes in reaction energies are of the order of 1 kcal/mol.

The XPS calculations were performed using the final-state approximation by exciting half of an electron to a virtual orbital. The excited electrons are then allowed to relax upon removal of the core hole, and all nonexcited core electrons remain frozen. Each atom is excited in an independent calculation. Only the relative values of core level binding energies obtained through this method are reliable.^68−70^

## Supporting Information Available

Content includes additional NMR, XPS, UPS, IR, CV, AFM, contact angle, DFT calculations, and details about coverage calculations (PDF)
