# Supplementary material for: Strong Substrate–Adsorbate Interactions Direct the Impact of Fluorinated N-Heterocyclic Carbene Monolayers on Au Surface Properties
Source: ACS Appl Mater Interfaces. 2024 Nov 18;16(47):65469–79. doi: 10.1021/acsami.4c12514 (PMC11615852; doi:10.1021/acsami.4c12514)
Supplement: Supplementary file 1 — am4c12514_si_001.pdf [file am4c12514_si_001.pdf]

Supporting Information

Strong Substrate-Adsorbate Interactions Direct the Impact of Fluorinated N-heterocyclic Carbene Monolayers on Au Surface Properties

Iris Berg[1,2+], Rajarshi Mondal[1+], Joshua M. Sims[3], Tzipora Ben-Tzvi[1,2], Linoy Lahav[1,2], Barak Friedman[1,2], Carine Michel[3], Zackaria Nairoukh[1]* and Elad Gross[1,2]*

1 Institute of Chemistry, The Hebrew University, Jerusalem 91904, Israel

2 The Center for Nanoscience and Nanotechnology. The Hebrew University. Jerusalem 91904. Israel

3 ENSL, CNRS, Laboratoire de Chimie UMR 5182, 46 allée d'Italie, F69364 Lyon France

+ These authors contributed equally to this work

Corresponding authors

* Elad Gross: elad.gross@mail.huji.ac.il

* Zackaria Nairoukh: z.nairoukh@mail.huji.ac.il

Table S1: Area quantification calculations

For NO_2 functionalized NHCs:

N1s/Au4f = 0.005

Surface density= $3.84*10^{-12}$ mol/cm^2

In the NO_2 functionalized NHCs, there are 6 nitrogen atoms, whereas in the fluorinated NHCs there are 2, so that the value was divided by 3.

For the value based on the XPS data, the calculation was performed using equation S1: [1]

(Eq. S1) $d = \frac{\frac{N1s}{Au4f} * 1.19 * 10^{16} cm^{-2}}{2\frac{nitrogen\ atoms}{molecule} * N_A}$

Molecule	N1s/Au4f	Surface Density (mol / cm^2) based XPS and electroreduction of NO_2 functionalized NHC	Surface Density (mol / cm^2)- based on XPS
Mono-F	0.0022	5.0E-12	2.2E-11
2,4-diF	0.0022	4.9E-12	2.1E-11
3,5-diF	0.0027	6.3E-12	2.7E-11
2,6-diF	0.0027	6.2E-12	2.6E-11
Tri-F	0.0032	7.3E-12	5.9E-11
Tetra-F	0.0062	1.4E-11	6.1E-11
Penta-F	0.0078	1.8E-11	7.7E-11

Table S2: Calculated binding energies (eV), dihedral angles and altitude of Au atom for NHCs with phenyl substituents and for the various F-NHCs.

**Name**	**Adsorption energy (eV)**	**Dihedral angle prior adsorption (°)**	**Dihedral angle after adsorption (°)**	**Au altitude (Å)**
Phenyl rings	-2.55	-90.6	-138.9	1.57
Mono-F	-2.55	-90.3	-143.3	1.58
2.4-diF	-2.10	-138.7	-133.6	1.44
3,5-diF	-2.18	-90.0	-140.9	1.72
2,6-diF	-2.24	-90.7	-127.6	1.50
Tri-F	-2.06	-89.8	-131.2	1.52
Tetra-F	-1.92	-90.2	132.2	1.38
Penta-F	-1.93	-90.9	134.0	1.38

Table S3: F1s XPS data of F-NHCs obtained by DFT for the tetra-F NHC in different states of decomposition (remove-X means the F-XPS of the NHC from which X has been removed. X means the F-XPS of substituent X). The relative bands show the energy level of the predicted XPS bands relative to the non-decomposed tetra-F.

Molecule	atom	1s	Relative bands
tetra	F1	-698.3792	0.001
	F2	-698.2275	0.1527
	F3	-698.3802	0
	F4	-698.3759	0.0043
remove Me	F1	-698.317	0.0632
	F2	-698.3041	0.0761
	F3	-698.3174	0.0628
	F4	-698.3069	0.0733
remove F1	F2	-697.1332	1.247
	F3	-698.3657	0.0145
	F4	-698.1841	0.1961
remove F2	F1	-697.1189	1.2613
	F3	-698.3301	0.0501
	F4	-698.1416	0.2386
remove F3	F1	-698.2358	0.1444
	F2	-697.8869	0.4933
	F4	-697.3182	1.062
remove F4	F1	-698.0013	0.3789
	F2	-698.1155	0.2647
	F3	-697.1634	1.2168
Ph	F1	-697.3945	0.9857
	F2	-697.9931	0.3871
	F3	-698.012	0.3682
	F4	-697.4381	0.9421
F	F1	-692.9158	5.4644
remove H1	F1	-697.8974	0.4828
	F2	-698.0387	0.3415
	F3	-698.102	0.2782
	F4	-698.0388	0.3414
remove H2	F1	-698.3621	0.0181
	F2	-698.2844	0.0958
	F3	-698.3078	0.0724
	F4	-698.3449	0.0353
remove H3	F1	-698.3598	0.0204

	F2	-698.2814	0.0988
	F3	-698.292	0.0882
	F4	-698.317	0.0632
remove H4	F1	-698.3562	0.024
	F2	-698.2812	0.099
	F3	-698.3052	0.075
	F4	-698.3346	0.0456
remove H5	F1	-698.365	0.0152
	F2	-698.277	0.1032
	F3	-698.3026	0.0776
	F4	-698.3365	0.0437
remove H6	F1	-697.9371	0.4431
	F2	-697.1911	1.1891
	F3	-697.1621	1.2181
	F4	-697.8257	0.5545
Ad-Atom	F8	-698.4675	0.0132
	F9	-698.2178	0.2629
	F11	-698.4659	0.0148
	F12	-698.4807	0

Table S4: F1s and N1s XPS data of F-NHCs obtained by DFT for the different NHCs, in their original state and after breaking the C-N bond (NHC-Name-split). The relative bands show the energy level of the predicted XPS bands relative to the non-decomposed tetra-F.

Molecule	F n°	1s	Relative bands
2-4-diF_bis	5	-698.1043	0
	7	-698.0392	0.0651
	12	-697.9868	0.1175
	14	-698.0632	0.0411
2-4-diF_bis-split	5	-697.2444	0.8599
	7	-697.7395	0.3648
	9	-697.9813	0.123
	11	-698.0876	0.0167
2-6-diF	5	-698.0838	0.0049
	9	-698.0816	0.0071
	10	-698.0584	0.0303
	14	-698.0395	0.0492
2-6-diF-split	5	-698.0867	0.002
	7	-698.0696	0.0191
	8	-698.0555	0.0332
	10	-698.0887	0
3-5-diF	6	-698.1452	0
	8	-697.6947	0.4505
	11	-697.6907	0.4545
	13	-698.1082	0.037
3-5-diF-split	5	-697.5348	0.6104
	7	-697.4118	0.7334
	9	-697.7374	0.4078
	11	-697.8882	0.257
Mono-F	7	-697.8738	0.0177
	12	-697.8333	0.0582
mono-F-split	part7	-697.8915	0
	part2	-697.7636	0.1279
penta	8	-698.2867	0.0725
	9	-698.2069	0.1523
	10	-698.2832	0.076
	11	-698.3592	0
	12	-698.3181	0.0411
penta-split	5	-697.282	1.0772
	6	-697.947	0.4122

	7	-698.2439	0.1153
	8	-697.9791	0.3801
	9	-697.2331	1.1261
tri-diF	5	-698.2472	0.0846
	7	-698.0783	0.2535
	9	-698.0716	0.2602
	10	-698.0513	0.2805
	12	-698.039	0.2928
	14	-698.2161	0.1157
tri-F-split	5	-697.0225	1.3093
	7	-697.6224	0.7094
	9	-696.8326	1.4992
	10	-697.7309	0.6009
	12	-698.0997	0.2321
	14	-698.3318	0
tetra	8	-698.3792	0.001
	9	-698.2275	0.1527
	11	-698.3802	0
	12	-698.3759	0.0043
tetra-split	5	-697.2764	1.1038
	6	-697.8138	0.5664
	8	-697.8052	0.575
	9	-697.2207	1.1595

Table S5: Reaction energies, transition state energies, length of the breaking C-N bond at the transition state and experimental temperature at which decomposition was first observed. In particular the tetra-F NHC which was experimentally determined to be the least stable is shown here to have the lowest activation energy for C-N cleavage.

Name	$\Delta G^{\neq}$ kcal/mol	$d^{\neq}$ Å	Decomp. Temp °C
mono-F	48.9	2.03	100
2,4di-F	46.1	1.99	100
3,5 di-F	42.8	1.96	100
2,6 di-F	41.8	1.97	r.t.
tri-F	40.7	1.97	r.t
tetra-F	36.7	2.03	r.t.
penta-F	36.8	2.04	r.t-100

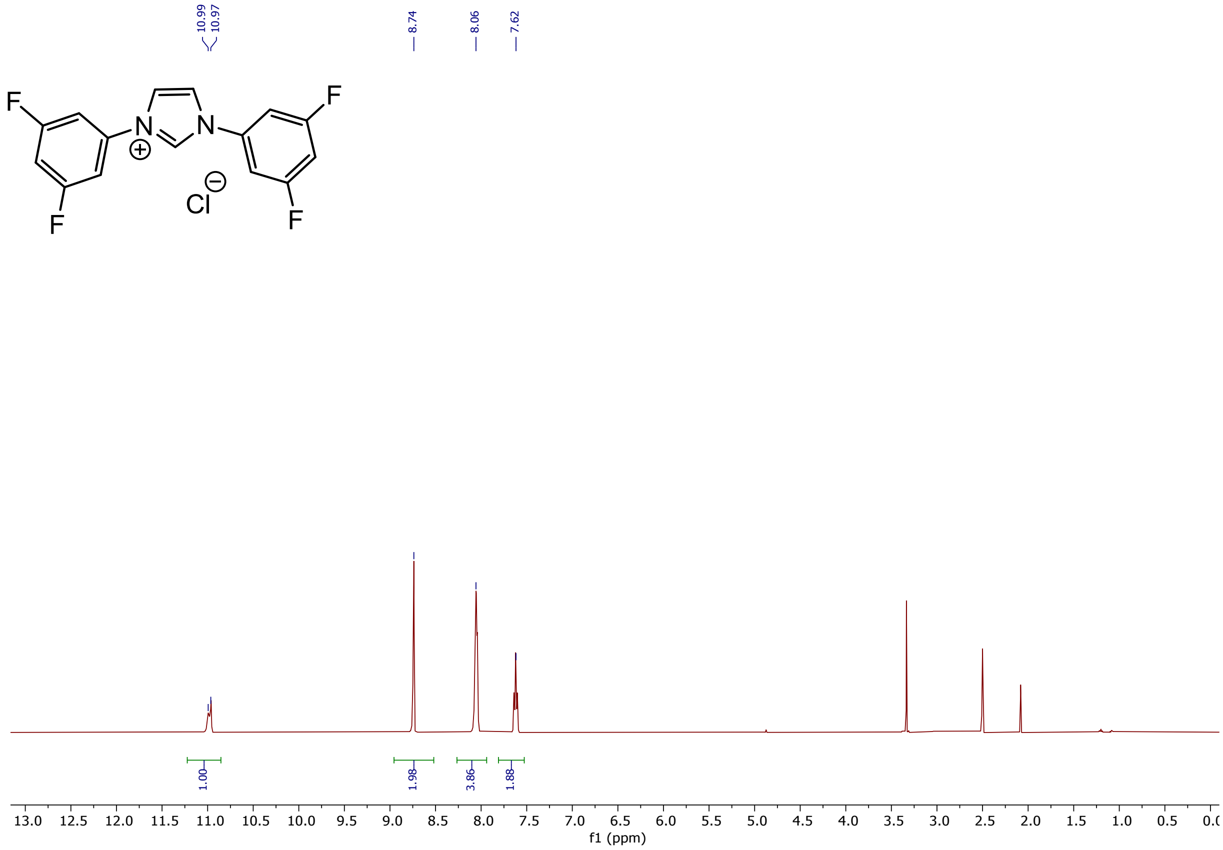

Figure S1: ^{1}H NMR of 3,5 di-F-Cl^{-} salt

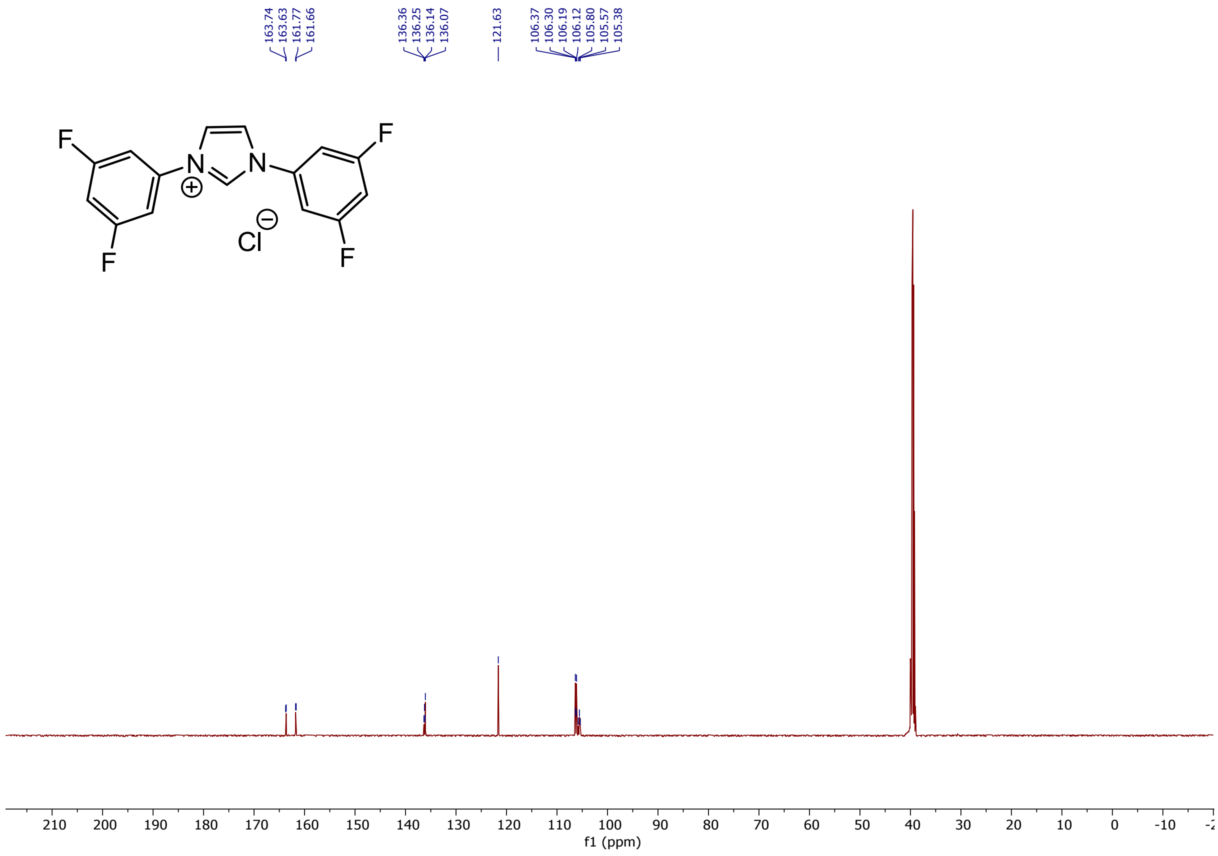

Figure S2: ^{13}C NMR of 3,5 di-F-Cl^- salt

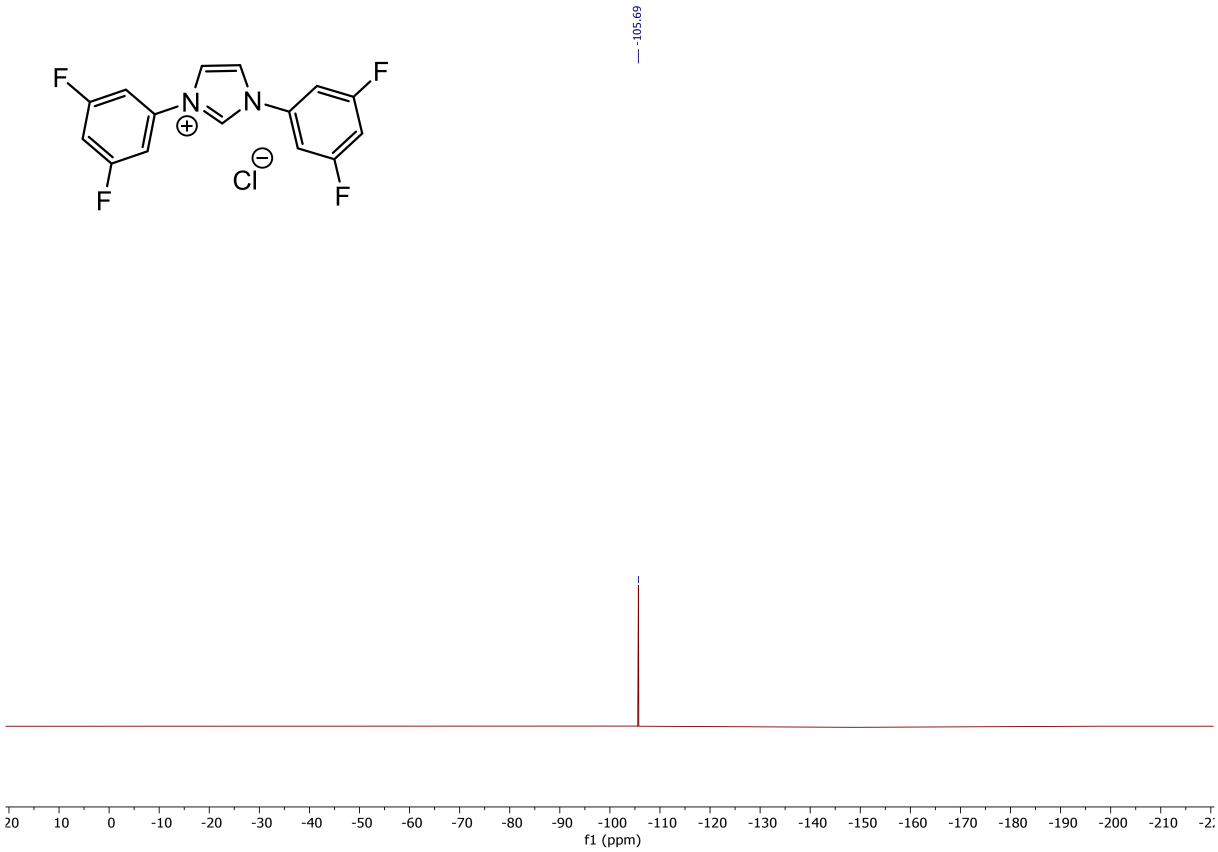

Figure S3: ^{19}F NMR of 3,5 di-F-Cl^- salt

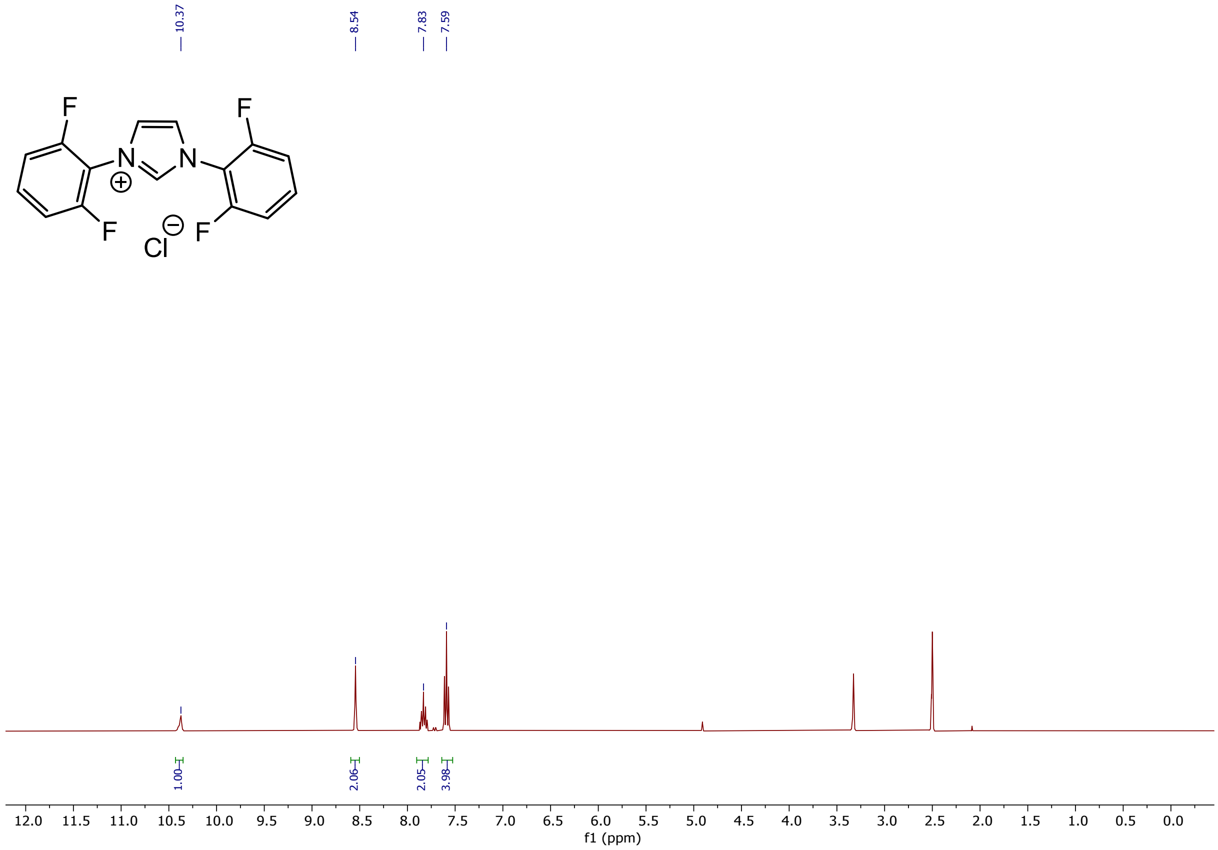

Figure S4: ^{1}H NMR of 2,6 di-F-Cl^{-} salt

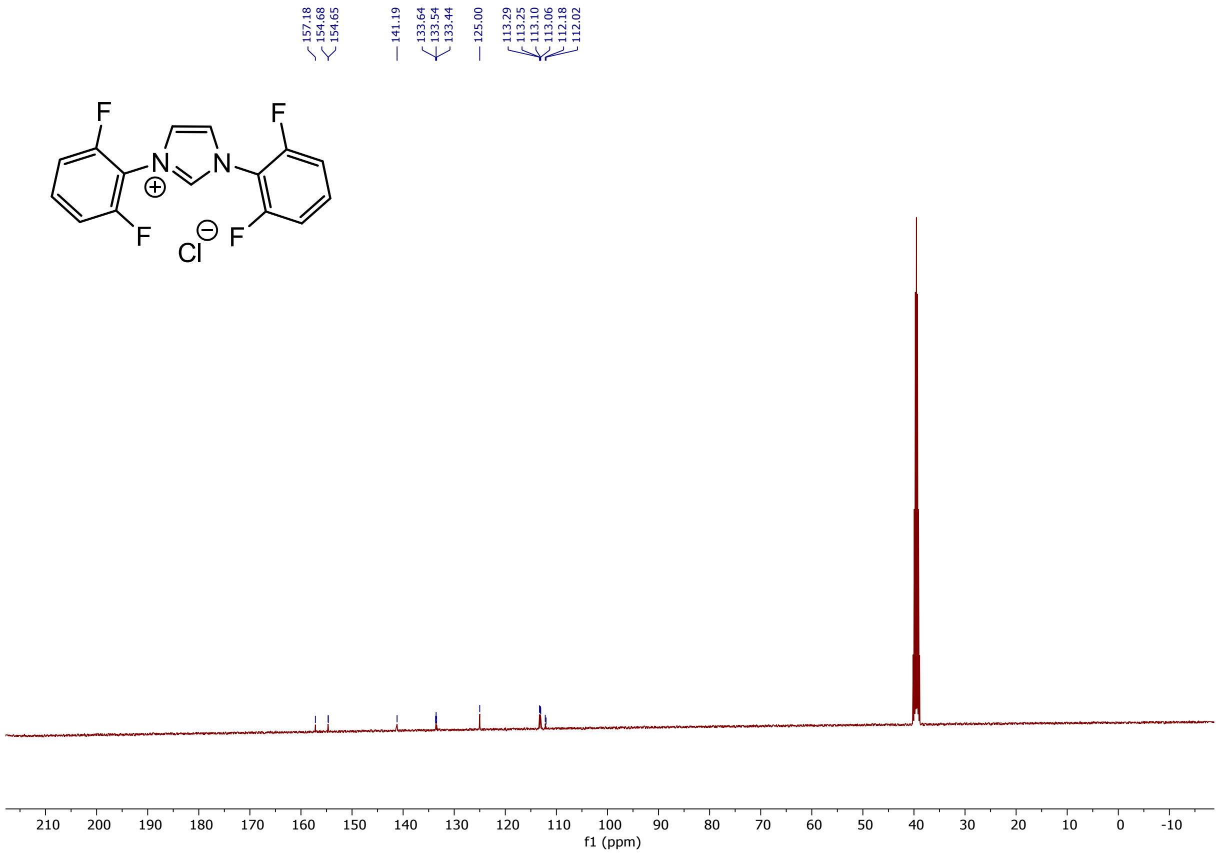

Figure S5: ^{13}C NMR of 2,6 di-F-Cl^{-} salt

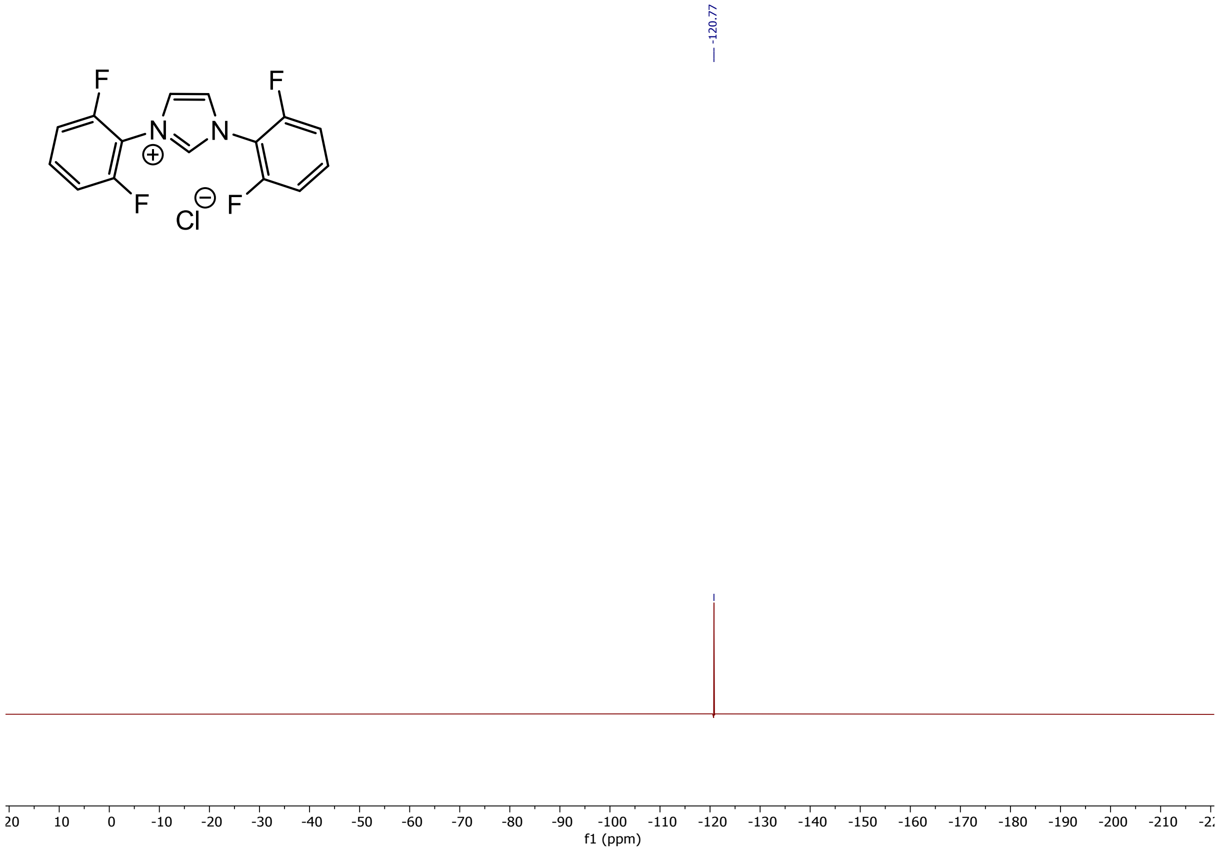

Figure S6: ^{19}F NMR of 2,6 di-F-Cl^- salt

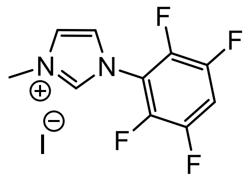

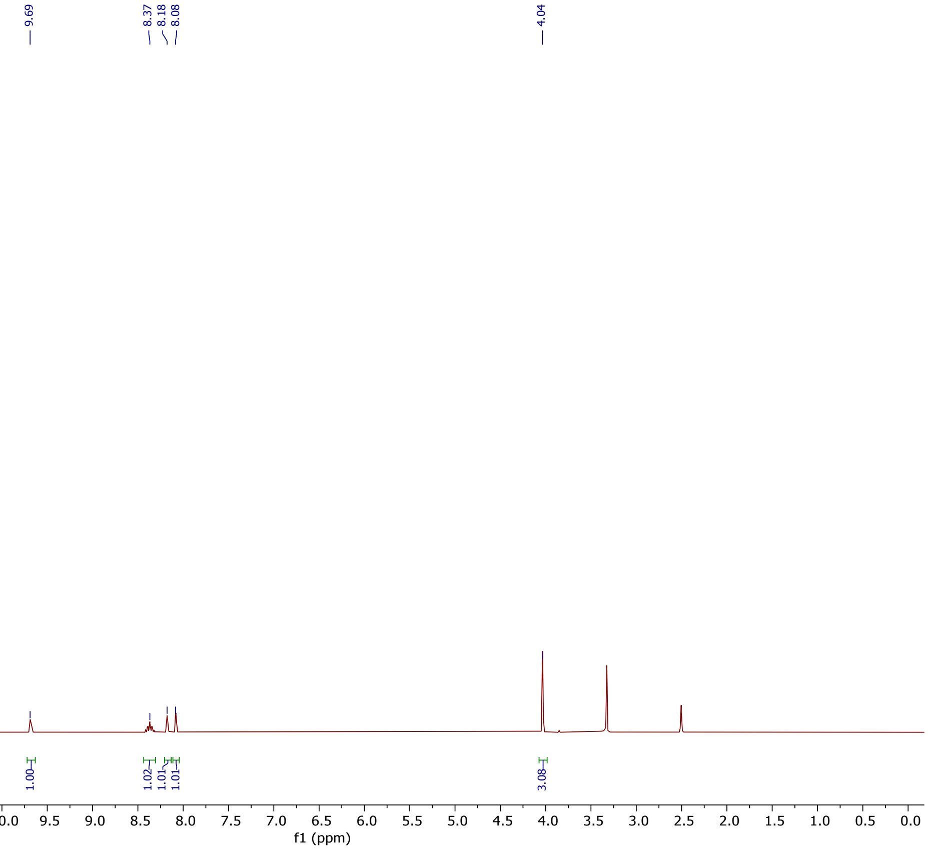

Figure S7: ^{1}H NMR of tetra-F-I^{-} salt

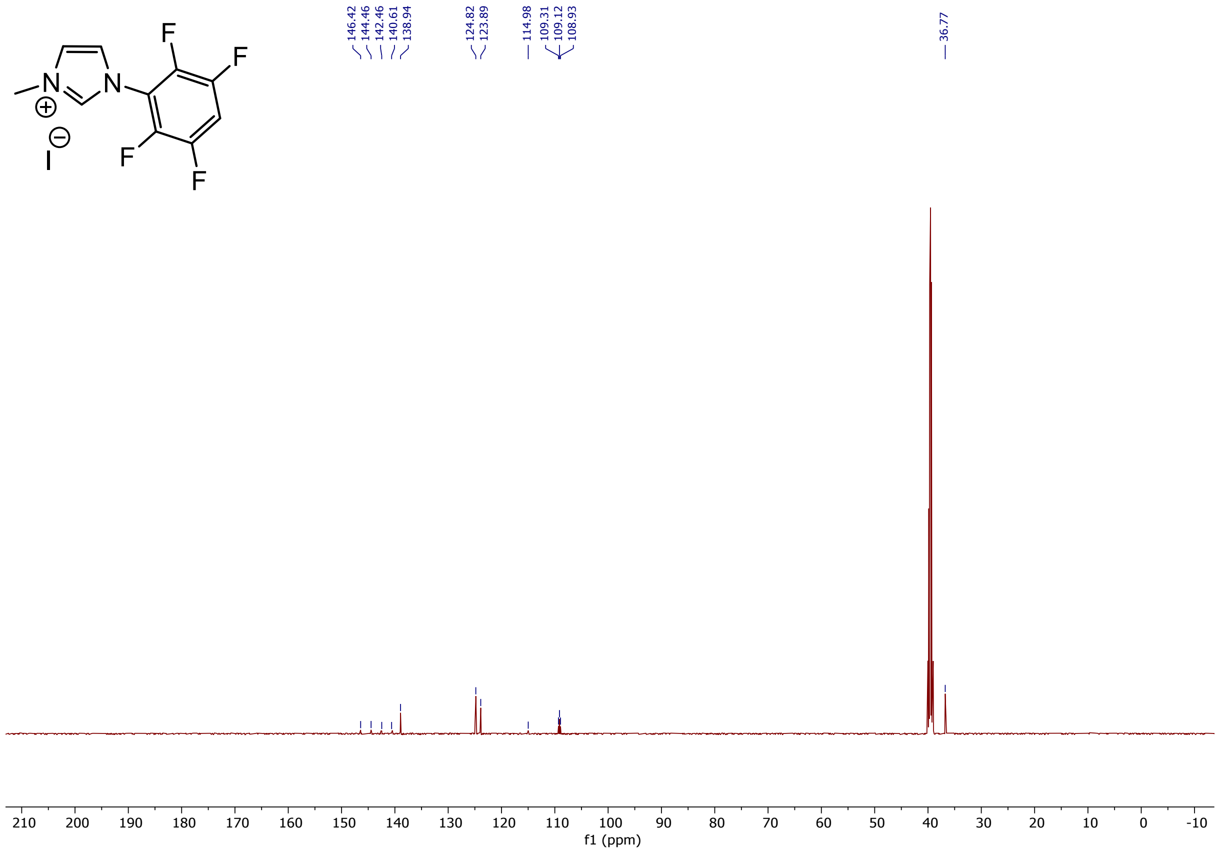

Figure S8: ^{13}C NMR of tetra-F-I$^-$ salt

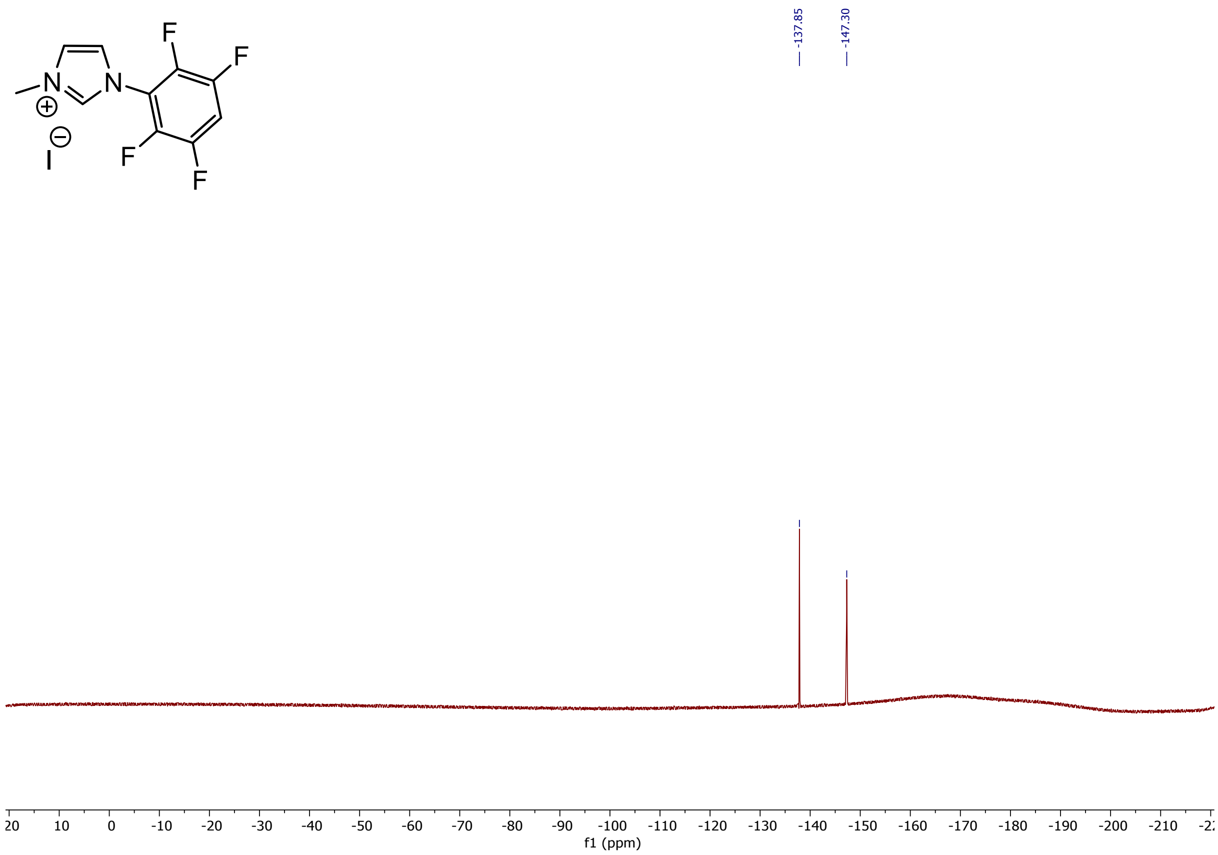

Figure S9: ^{19}F NMR of tetra-F-I$^-$ salt

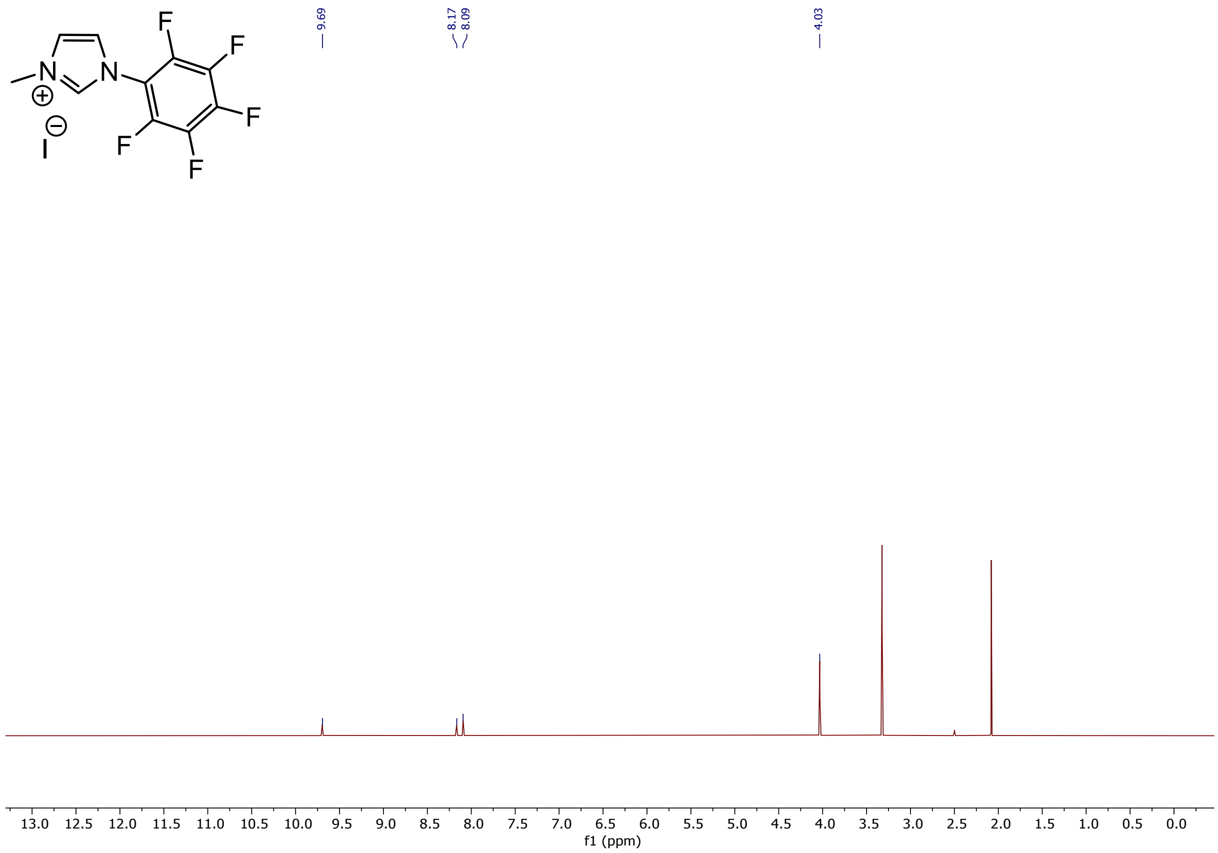

Figure S10: ^{1}H NMR of penta-F-I^{-} salt

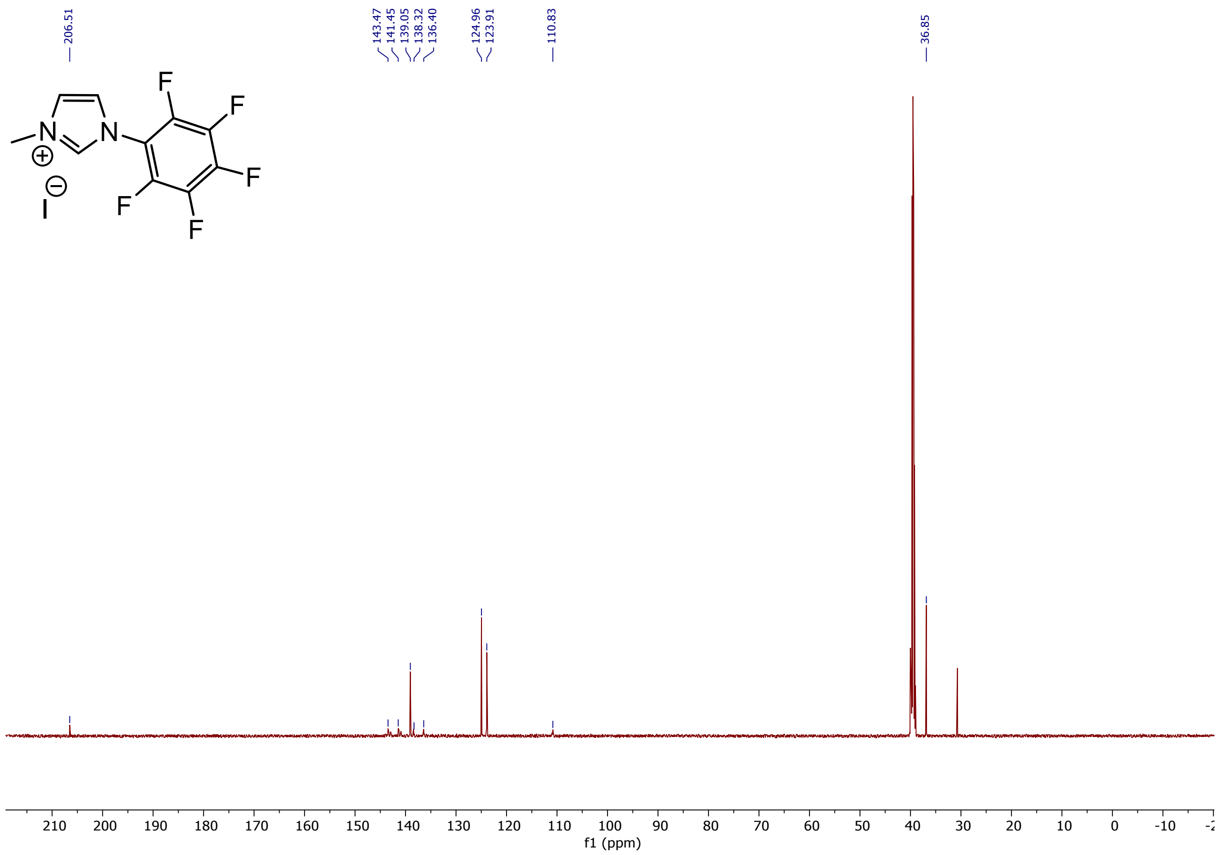

Figure S11: ^{13}C NMR of penta-F-I$^-$ salt

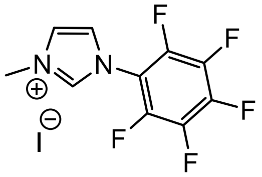

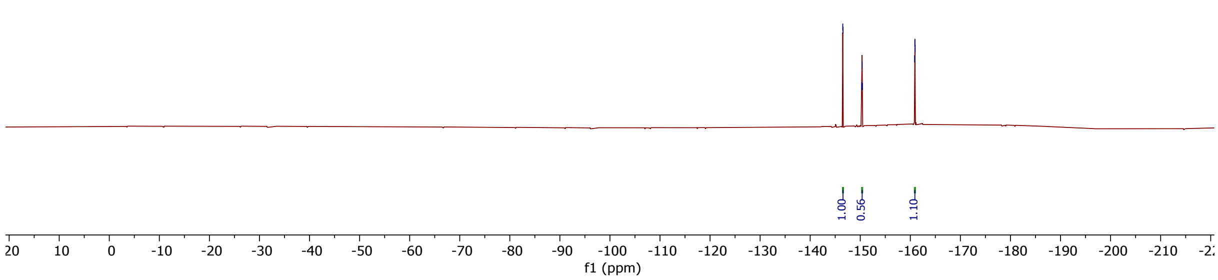

Figure S12: ^{19}F NMR of penta-F-I^- salt

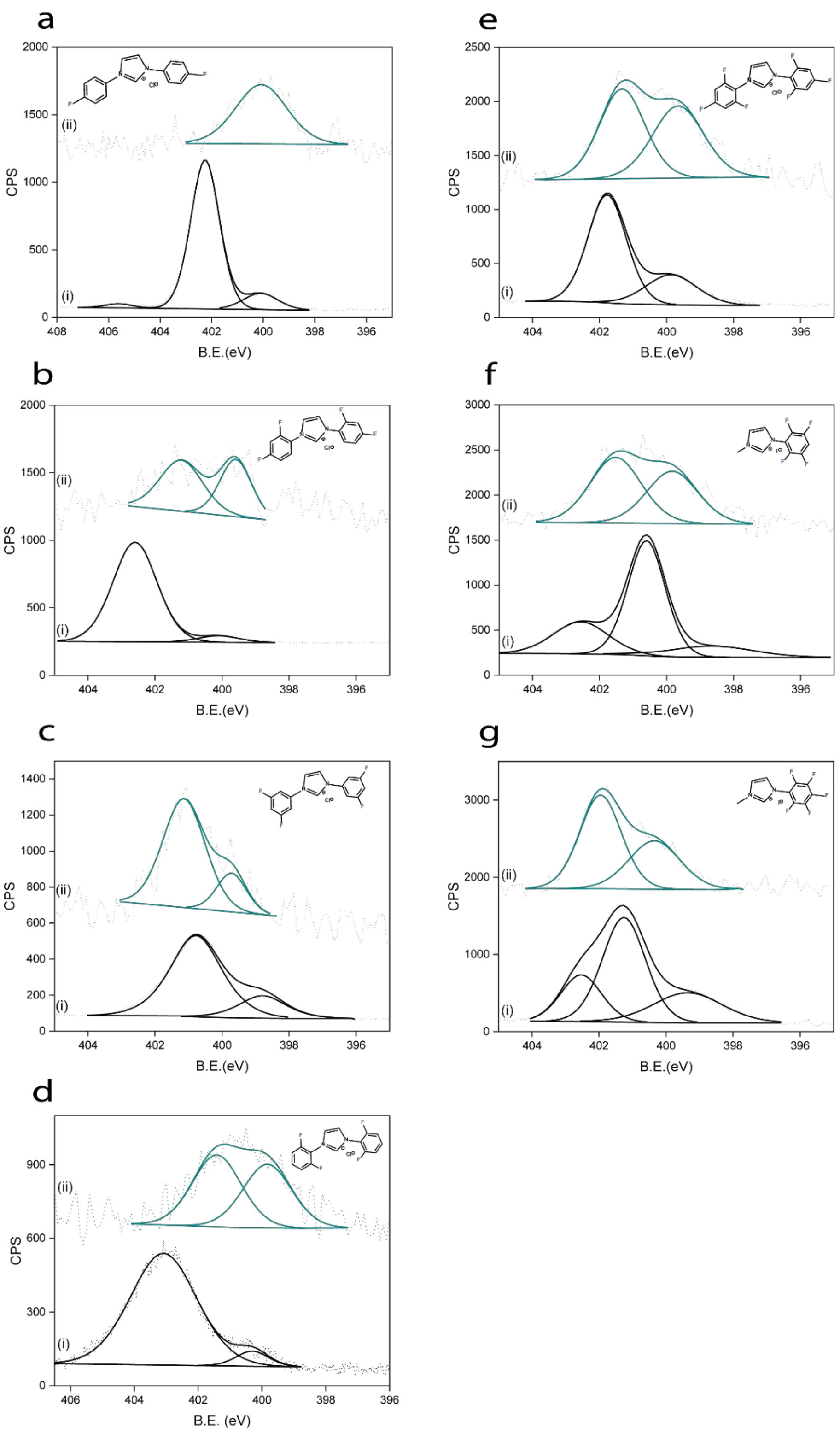

Figure S13: N1s XP-spectra of (i) imidazolium salt precursor and (ii) surface-anchored F-NHC on Au (111): (a) mono-fluorinated (b) 2,4 difluorinated (c) 3,5 difluorinated (d) 2,6 difluorinated (e) tri-fluorinated (f) asymmetric tetra-fluorinated and (g) asymmetric penta-fluorinated NHCs.

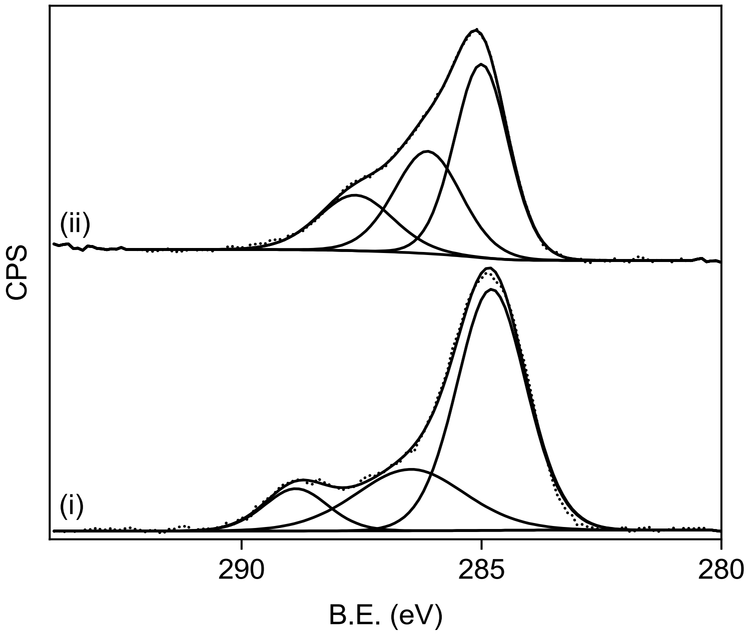

Figure S14: C1s XP-spectra of (i) a bare gold film, cleaned in the same method as the samples used for the manuscript and (ii) a gold film coated with 2,4 difluorinated NHC.

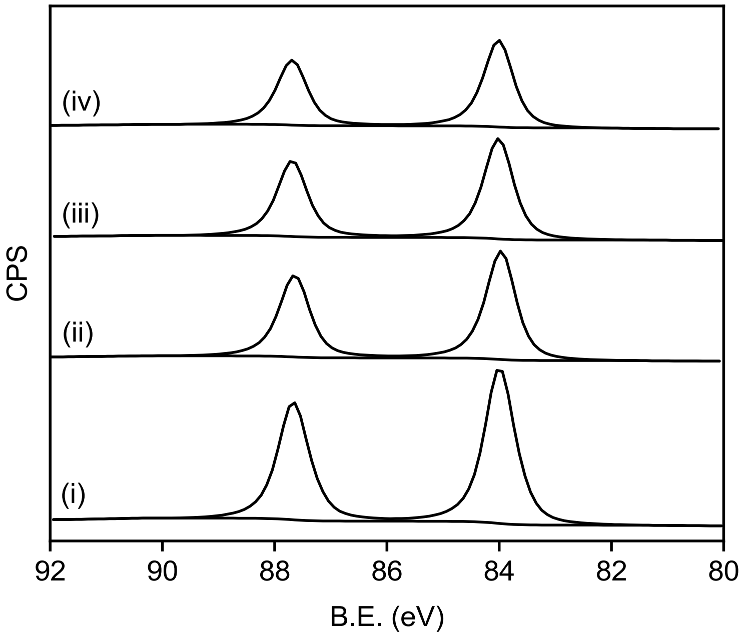

Figure S15: Au4f XP-spectra of bare Au (i), and of mono-F (ii), tri-F (iii), and penta-F (iv) NHCs coated Au. F-NHC deposition did not result any noticeable changes in the Au4f XPS pattern.

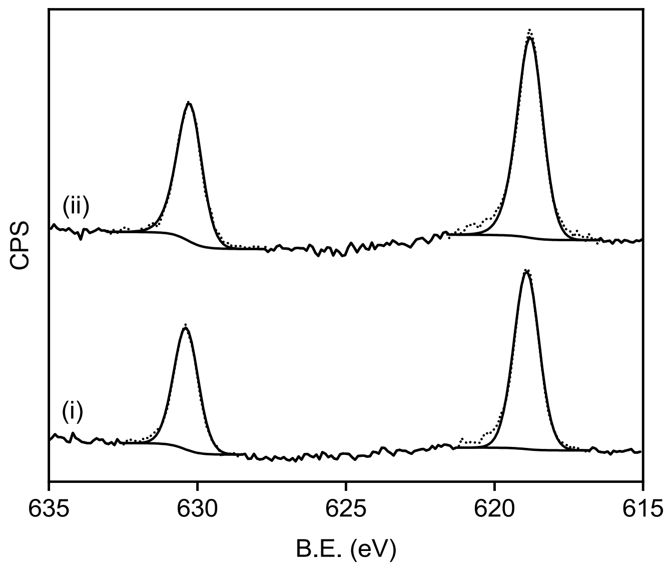

Figure S16: I3d XP-spectra of tetra-F (i) and penta-F (ii) NHCs coated Au surfaces.

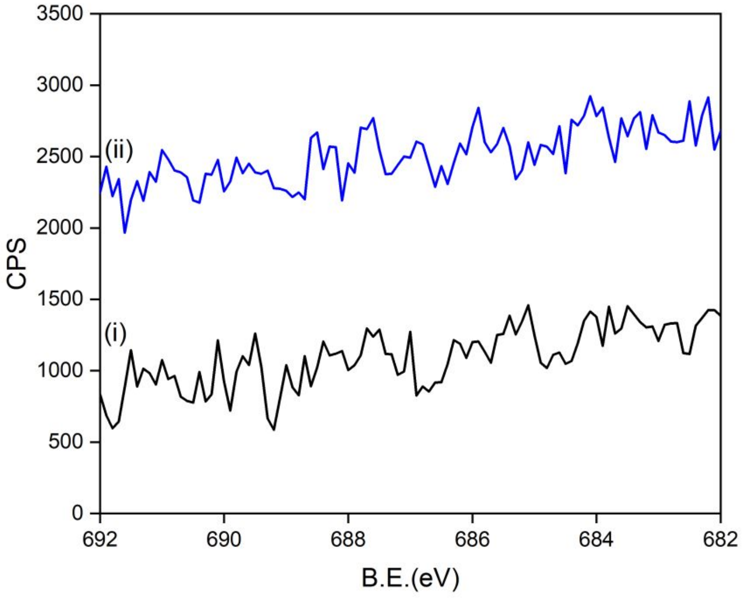

Figure S17: F1s XP-spectra of imidazolium salt that was drop-casted using a solution of 100mM (i) tetra-fluorobenzene and (ii) penta-fluorobenzene.

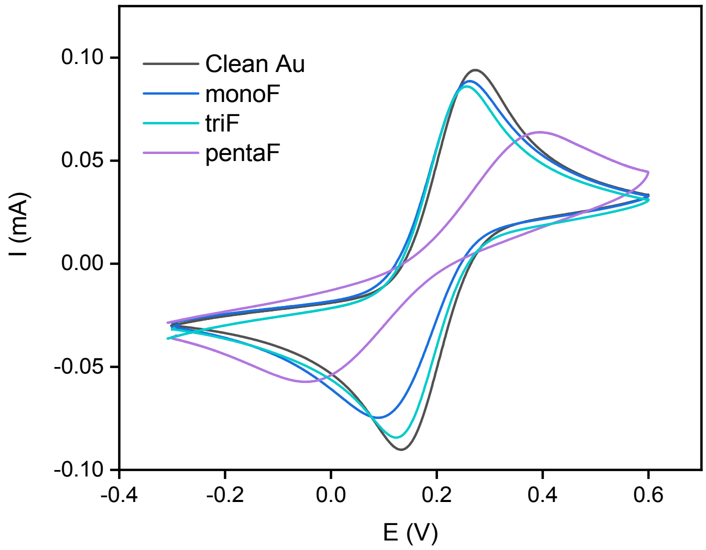

Figure S18: Cyclic voltammograms of 10 mM $[Fe(CN)_6]^{-3}/[Fe(CN)_6]^{-4}$ in 0.1 M KCl recorded with Au electrode before and following deposition of mono-, tri-, and penta-F NHCs.

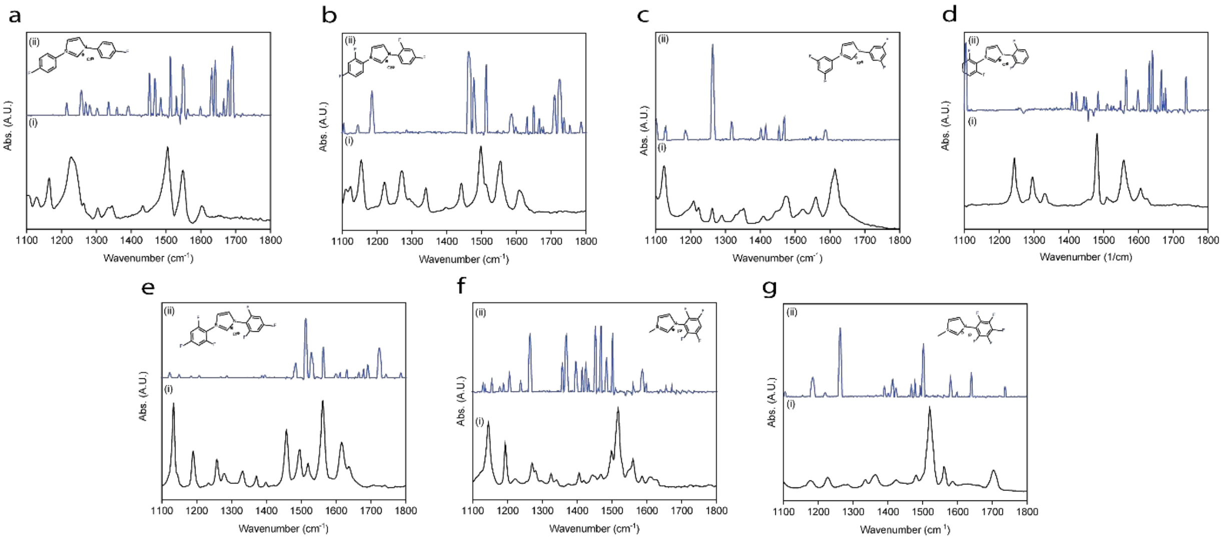

Figure S19: ATR (spectra i) and PM-IRRAS (spectra ii) of imidazolium salt precursors and surface-anchored F-NHCs, respectively: (a) mono-fluorinated (b) 2,4 difluorinated (c) 3,5 difluorinated (d) 2,6 difluorinated (e) trifluorinated (f) asymmetric tetrafluorinated and (g) asymmetric pentafluorinated NHCs.

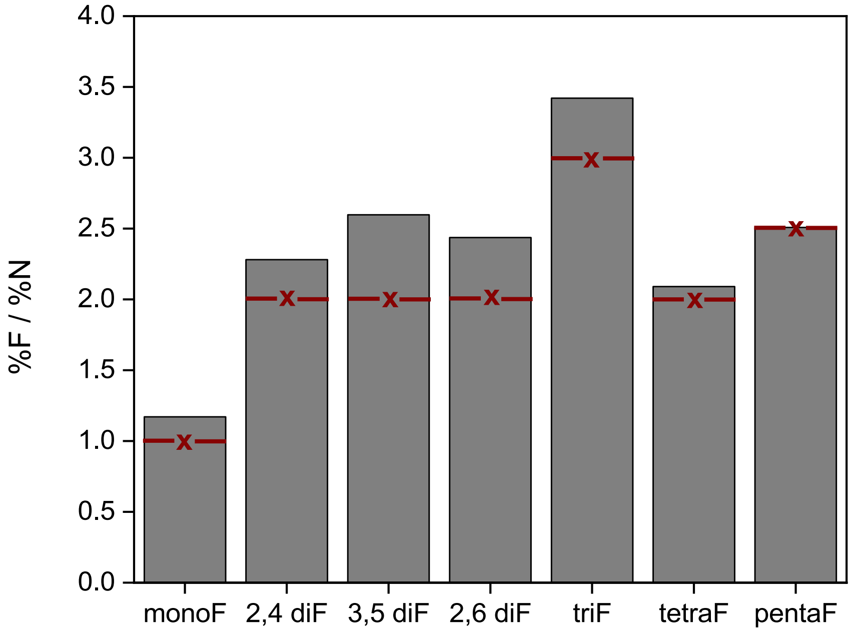

Figure S20: The atomic percentage of fluorine to nitrogen ratios for F-NHC precursors (grey-coloured bars), as determined by XPS measurements, relatively to the expected ratio (red-coloured lines).

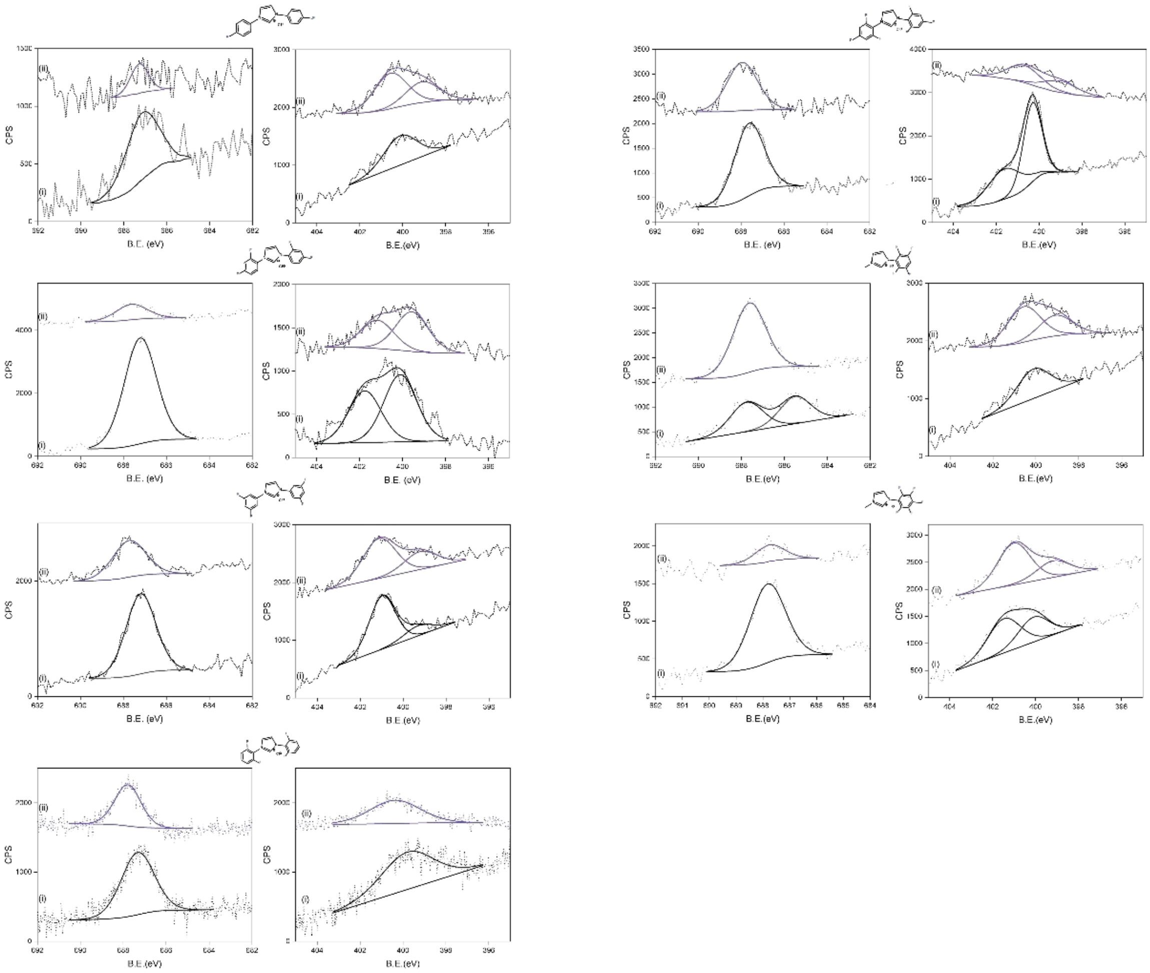

Figure S21: F1s (right panels) and N1s (left panels) XPS signals of F-NHCs following annealing to (i) 100 and (ii) 200 °C.

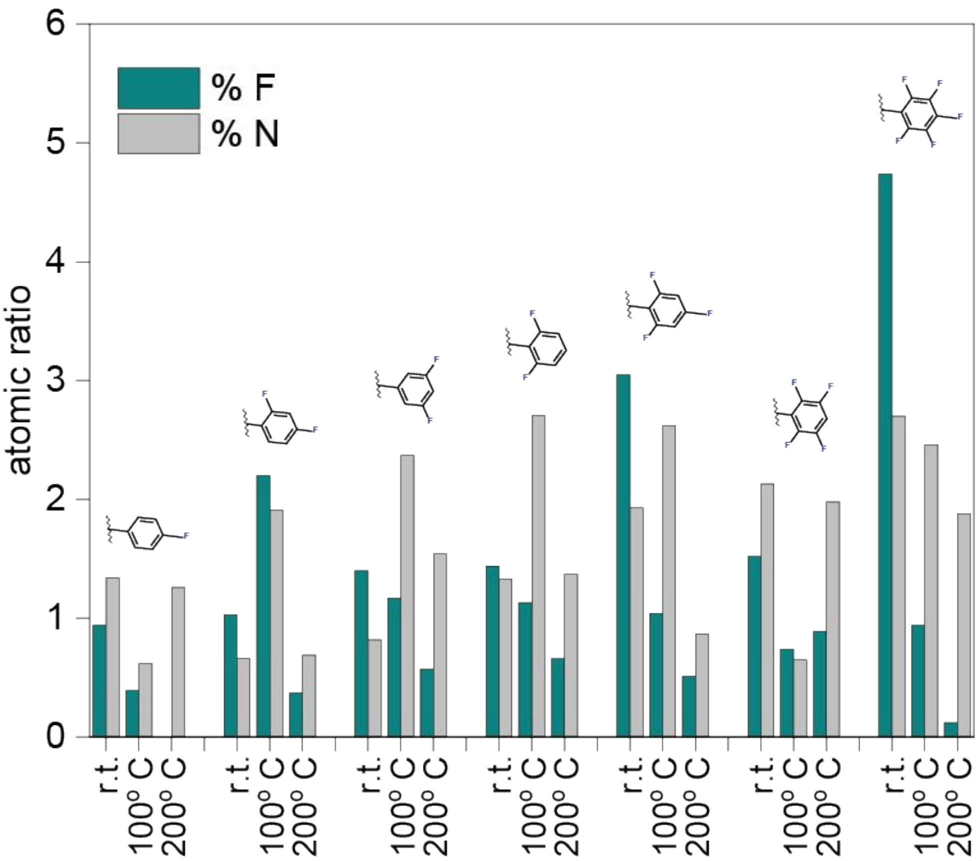

Figure S22: Atomic percentage of flour and nitrogen in self-assembled F-NHCs at room temperature and following annealing to 100 and 200 °C for 2h.

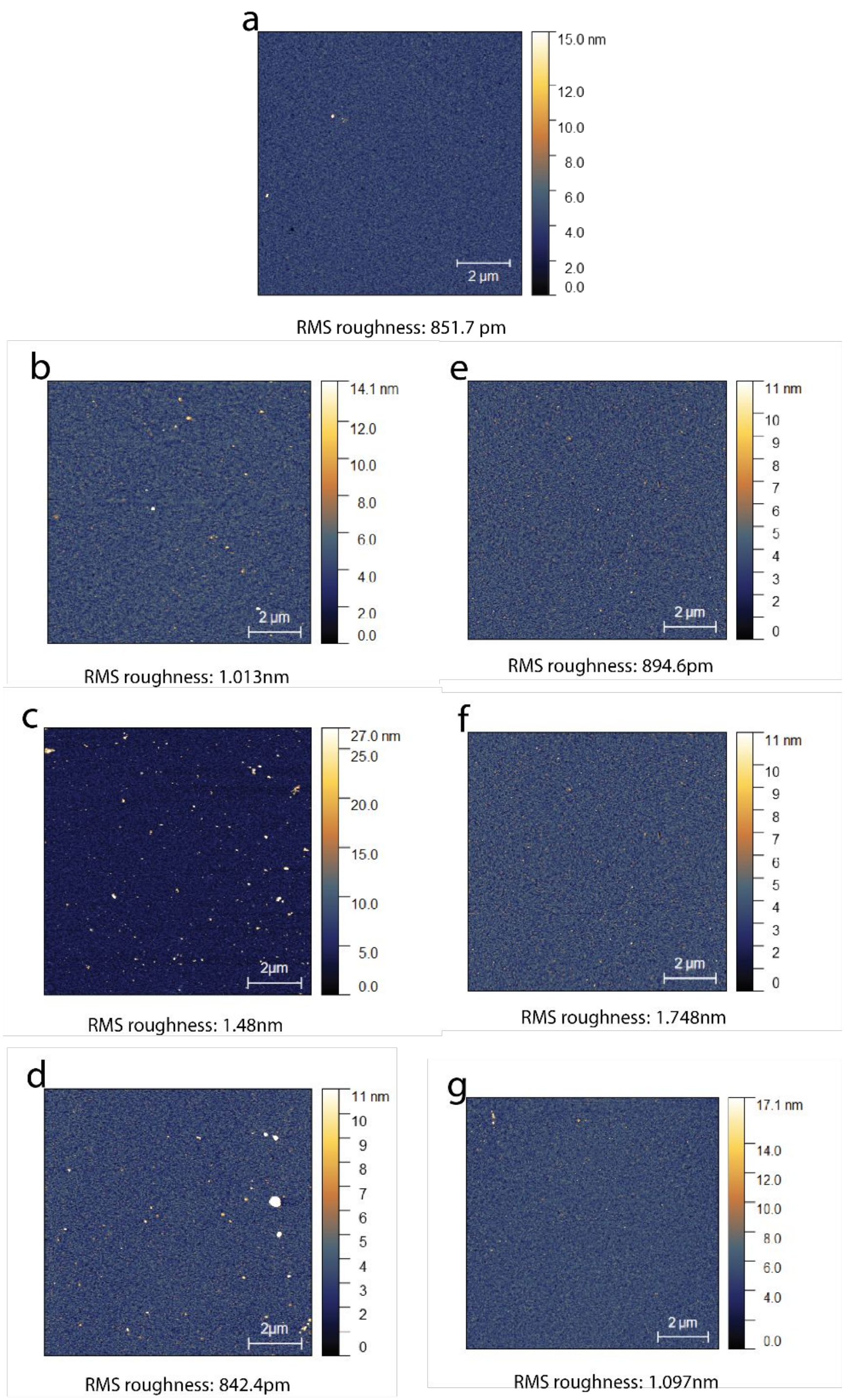

Figure S23: AFM topography and RMS roughness values of bare Au surface (a) and Au surface that was coated with mono- (b and e), tri- (c and f) and penta- (d and g) fluorinated NHCs. Au films were characterized before (b-d) and after (e-g) annealing to 100 °C for 2 h. The white dots that are detected following deposition are correlated to solvent or base residues from the deposition process, which desorbed upon annealing.

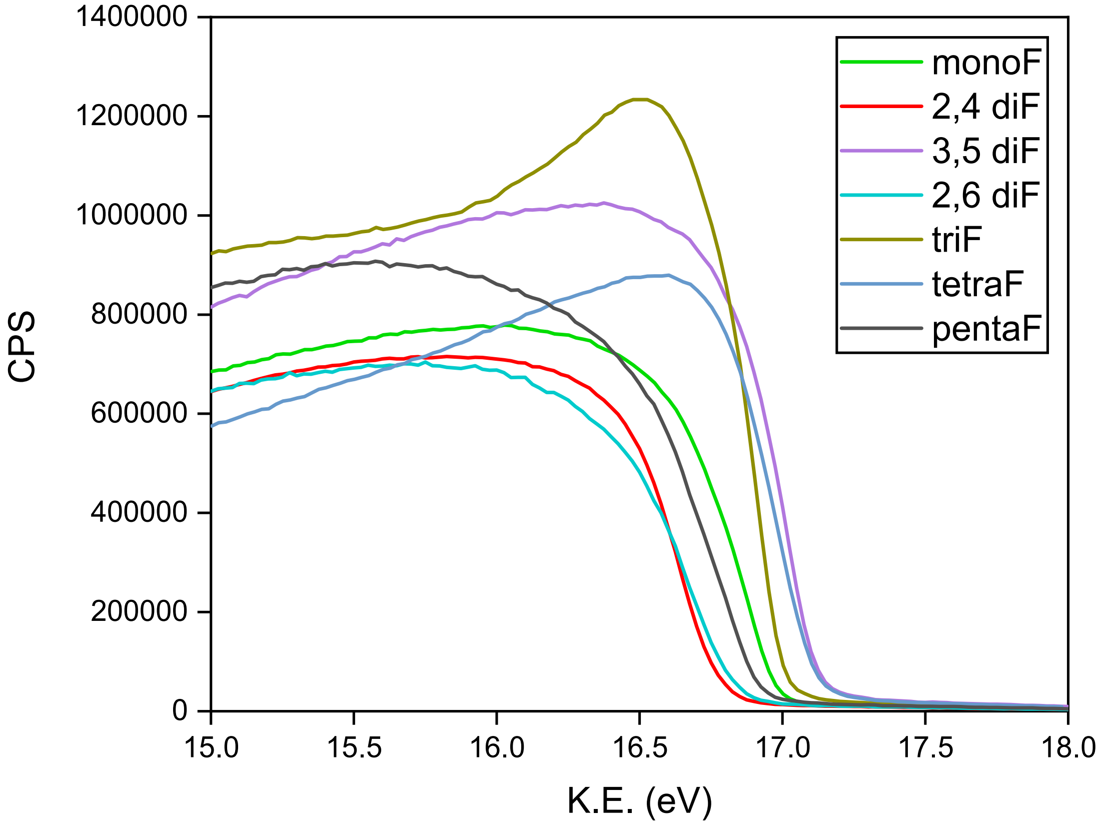

Figure S24: UPS signals for the F-NHCs on Au (111).

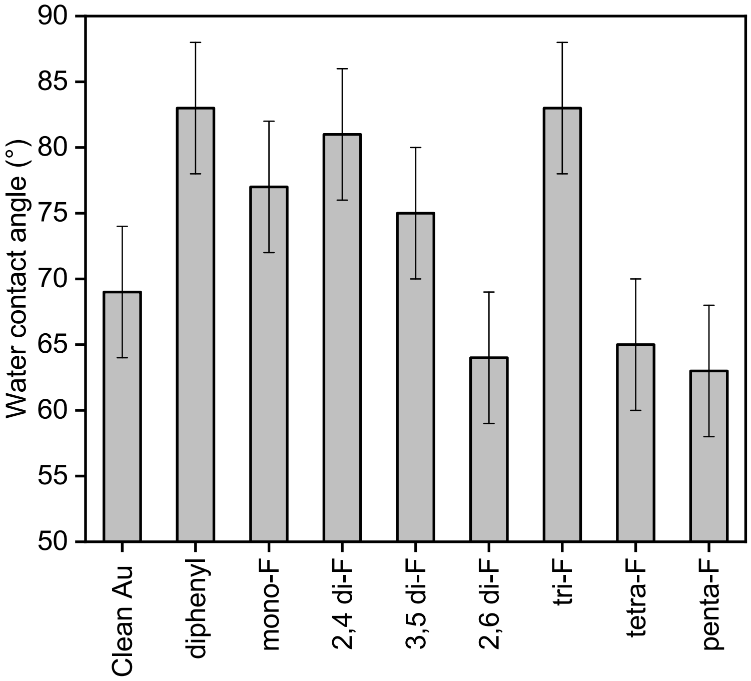

Figure S25: Water contact angle values measured for F-NHCs and diphenyl-NHC coated Au films. Error bars represent the standard deviation based on analysis of multiple measurements.

SI References

(1) Kim, H. K.; Hyla, A. S.; Winget, P.; Li, H.; Wyss, C. M.; Jordan, A. J.; Larrain, F. A.; Sadighi, J. P.; Fuentes-Hernandez, C.; Kippelen, B.; Brédas, J. L.; Barlow, S.; Marder, S. R. Reduction of the Work Function of Gold by N-Heterocyclic Carbenes. *Chem Mat* **2017**, *29* (8), 3403–3411. https://doi.org/10.1021/acs.chemmater.6b04213.
